# NirD curtails the stringent response by inhibiting RelA activity in *Escherichia coli*

**DOI:** 10.7554/eLife.64092

**Published:** 2021-07-29

**Authors:** Loïc Léger, Deborah Byrne, Paul Guiraud, Elsa Germain, Etienne Maisonneuve

**Affiliations:** 1 Laboratoire de Chimie Bactérienne, Institut de Microbiologie de la Méditerranée, CNRS-Aix Marseille Univ (UMR7283)MarseilleFrance; 2 Protein Expression Facility, Institut de Microbiologie de la Méditerranée, CNRS-Aix Marseille UnivMarseilleFrance; Harvard Medical SchoolUnited States; National Institute of Child Health and Human DevelopmentUnited States

**Keywords:** stringent response, RelA, NirD, (p)ppGpp, bacterial stress response, *E. coli*

## Abstract

Bacteria regulate their metabolism to adapt and survive adverse conditions, in particular to stressful downshifts in nutrient availability. These shifts trigger the so-called stringent response, coordinated by the signaling molecules guanosine tetra and pentaphosphate collectively referred to as (p)ppGpp. In *Escherichia coli*, accumulation of theses alarmones depends on the (p)ppGpp synthetase RelA and the bifunctional (p)ppGpp synthetase/hydrolase SpoT. A tight regulation of these intracellular activities is therefore crucial to rapidly adjust the (p)ppGpp levels in response to environmental stresses but also to avoid toxic consequences of (p)ppGpp over-accumulation. In this study, we show that the small protein NirD restrains RelA-dependent accumulation of (p)ppGpp and can inhibit the stringent response in *E. coli*. Mechanistically, our in vivo and in vitro studies reveal that NirD directly binds the catalytic domains of RelA to balance (p)ppGpp accumulation. Finally, we show that NirD can control RelA activity by directly inhibiting the rate of (p)ppGpp synthesis.

## Introduction

Bacteria have evolved numerous molecular mechanisms to detect and cope with environmental stress, including the use of nucleotide-based signaling pathways to efficiently coordinate cellular processes and provide a fast response. Among these signaling pathways, the stringent response is a general stress response that is mediated by the accumulation of the nucleotides guanosine 5′-diphosphate 3′-diphosphate (ppGpp) and guanosine 5′-triphosphate 3′-diphosphate (pppGpp), collectively known as (p)ppGpp (Potrykus and Cashel, 2008). These alarmones allow bacteria to rapidly respond and adapt to various conditions of nutritional and environmental stress by affecting gene expression and metabolism. In Gram-negative bacteria, (p)ppGpp binds RNA polymerase, thereby altering its promoter selectivity, which results in genome-wide transcriptional reprograming (Durfee et al., 2008; Ross et al., 2013; Ross et al., 2016; Sanchez-Vazquez et al., 2019; Traxler et al., 2008). Additionally, (p)ppGpp also binds directly and alters the activity of several enzymes including DNA primase, translation factors, lysine decarboxylase, and polyphosphate kinase (Corrigan et al., 2016; Kanjee et al., 2012; Wang et al., 2019a; Zhang et al., 2018). Importantly, this rewiring of cell physiology also appears to play a critical role in the regulation of bacterial virulence, survival during host invasion, and antibiotic resistance and tolerance (Hauryliuk et al., 2015; Hengge, 2020; Irving et al., 2021; Potrykus and Cashel, 2008).

In *Escherichia coli*, (p)ppGpp synthesis and hydrolysis are regulated by RelA and SpoT, which are members of RelA/SpoT homologue (RSH) (Atkinson et al., 2011) enzymes and share similar domain architecture. The enzymatic N-terminal half (NTD) has the synthetase (SYN) and the hydrolase (HYD) domains, and the C-terminal half (CTD) of the protein contains conserved domains, which play a critical role in sensing and transducing stress signals to the catalytic domains (Gratani et al., 2018; Hauryliuk et al., 2015; Hogg et al., 2004; Mechold et al., 2002; Pausch et al., 2020; Takada et al., 2020; Tamman et al., 2020). SpoT is a bifunctional enzyme with both hydrolytic and synthetic activities while RelA is a monofunctional (p)ppGpp synthetase. Indeed, despite close sequence similarity between RelA and SpoT, RelA maintains a ‘pseudo’-hydrolase domain that is structurally conserved but enzymatically inactive. The regulation of RelA and SpoT activities represents an obvious checkpoint for (p)ppGpp levels which reveals many complexities (reviewed in Irving and Corrigan, 2018; Ronneau and Hallez, 2019).

Activation of the (p)ppGpp synthetase RelA occurs via a ribosomal mechanism in response to amino acid starvation or other stressful conditions, which ultimately result in amino acid starvation, for example, fatty acid starvation, that leads to lysine starvation (Haseltine and Block, 1973; Sinha et al., 2019). Under this condition, deacylated tRNAs accumulate and RelA interacts with uncharged tRNA at the vacant ribosomal A-site causing the activation of its (p)ppGpp synthetic activity (Arenz et al., 2016; Brown et al., 2016; Loveland et al., 2016; Winther et al., 2018). Inversely, RelA is suggested to be inhibited by intra- and/or inter-molecular interactions under replete conditions (Gropp et al., 2001; Turnbull et al., 2019; Yang and Ishiguro, 2001), even though it retains residual activity (Murray and Bremer, 1996). Importantly, the hydrolysis function of SpoT provides an opposing activity that is crucial for balancing the basal activity of RelA. Indeed, disruption of the *spoT* gene results in a toxic accumulation of (p)ppGpp and is therefore lethal (Xiao et al., 1991).

The regulation of SpoT activities involves sources of nutritional stress other than amino acid starvation. These include the deficiency of fatty acid (Seyfzadeh et al., 1993), phosphate (Spira et al., 1995), carbon (Xiao et al., 1991), and iron (Vinella et al., 2005). Interaction of SpoT with other factors seems to regulate the catalytic balance between the synthetic and hydrolytic activities. During fatty acid starvation, the accumulation of (p)ppGpp required a specific interaction between the acyl carrier protein and SpoT (Battesti and Bouveret, 2006). Similarly, the binding of the protein YtfK with the catalytic domains of SpoT triggers (p)ppGpp accumulation during phosphate or fatty acid starvation (Germain et al., 2019). In addition, stimulation of the SpoT hydrolase activity is driven through direct interaction with the anti-σ^70^ factor Rsd upon carbon downshift (Lee et al., 2018).

Given the importance of (p)ppGpp in stress survival, virulence, and antibiotic tolerance, we have designed a genetic assay for the identification of new protein candidates that can modulate (p)ppGpp homeostasis in *E. coli*. We thereby discovered that overproduction of NirD, the small subunit of the nitrite reductase, decreases intracellular levels of (p)ppGpp. Importantly, our results show that overexpression of *nirD* limits RelA-dependent accumulation of (p)ppGpp in vivo and can prevent activation of the stringent response during amino acid starvation. Moreover, our results show that NirD is not involved in (p)ppGpp breakdown. Rather we convincingly show that, mechanistically, NirD inhibits the (p)ppGpp synthetase activity of RelA through a specific and direct interaction with the catalytic domains of RelA in vivo and in vitro.

## Results

### Identification of genes counteracting the toxicity of (p)ppGpp over-accumulation mediated by RelA

**Figure 1.**
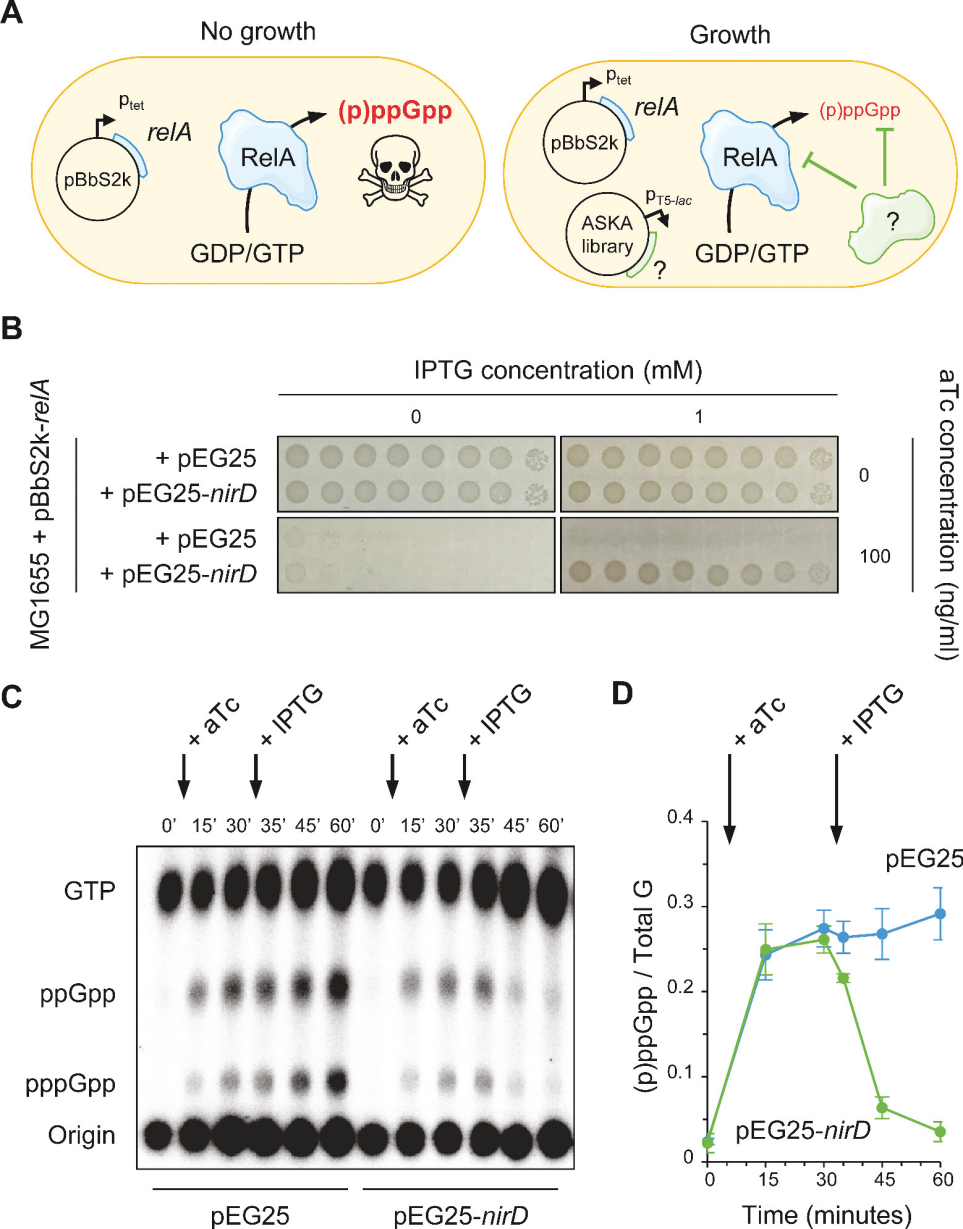
NirD can limit the RelA-dependent accumulation of (p)ppGpp. (**A**) Schematic representation of the genetic assay using ASKA library to identify genes that in multiple copies would suppress the growth defect associated with toxic over-accumulation of (p)ppGpp. Question marks denote a putative candidate that limits RelA-dependent accumulation of (p)ppGpp. (**B**) NirD suppresses the growth defect associated with RelA overproduction. Wild-type *E. coli* MG1655 cells were co-transformed with the plasmid derivatives pBbS2k-*relA* and pEG25-*nirD* inducible by anhydrotetracycline (aTc) and isopropyl β-D-1-thiogalactopyranoside (IPTG), respectively. Serial dilutions of stationary-phase cultures were spotted on NA plates containing the indicated concentrations of inducers. Additional controls and inducer concentrations are shown in Figure 1—figure supplement 1A. The results are representative of three independent experiments with similar results. (**C, D**) In vivo (p)ppGpp dynamic in *E. coli* MG1655 cells carrying pBbS2k-*relA* and pEG25-*nirD* after consecutive expression of the *relA* and *nirD* genes using 100 ng/mL aTc and 1 mM IPTG, respectively. Nucleotides extracted from samples collected at the indicated times were separated by thin layer chromatography. The autoradiogram (**C**) is representative of three independent experiments, and the curves of the relative levels of (p)ppGpp (**D**) are represented as the means of the three independent experiments, the error bars depicting the SDs. Figure 1—source data 1.Raw autoradiogram. Figure 1—source data 2.Quantification of (p)ppGpp.The amount of (p)ppGpp was normalized to total amount of G nucleotides observed in each sample. Total G is the sum of GTP and (p)ppGpp detected. The source data are provided for the relative levels of (p)ppGpp in Figure 1D, which are represented as the means and SDs of three independent experiments.

At high concentrations, (p)ppGpp becomes a potent inhibitor of bacterial cell growth. Indeed, rising (p)ppGpp levels by overexpression of the *relA* gene has been shown to inhibit *E. coli* growth in amino acid-rich media (Schreiber et al., 1991). To gain further insights into (p)ppGpp homeostasis, we developed a screening assay to identify genes that in multiple copies could reverse this toxicity (Figure 1A). In this study, the toxic accumulation of (p)ppGpp is achieved by induction of the *relA* gene cloned in the low-copy-number plasmid pBbS2k under the control of the anhydrotetracycline (aTc)-inducible P_tet_ promoter (Figure 1A). *E. coli* MG1655 cells harboring the pBbS2k-*relA* were transformed with a pooled collection of plasmids obtained from the ASKA library containing almost all *E. coli* K-12 genes (Kitagawa et al., 2005), each cloned into the high-copy-number vector pCA24N downstream of the isopropyl β-D-1-thiogalactopyranoside (IPTG)-inducible P_T5-lac_ promoter (Figure 1A). This genetic screen led us to identify 88 clones that repetitively grow on non-permissive conditions. Sequence analysis of the pCA24N derivatives from these 88 clones enabled the identification of four different genes. The *spoT*, *gppA,* and *nirD* genes were predominant with each one accounting for almost a third of the genes carried by the sequenced plasmids (33.0, 36.4, and 29.5%, respectively) while *uhpA* was only identified once (1.1%). Our observations that *spoT* and *gppA* revert toxicity associated to (p)ppGpp accumulation is consistent with previous observations (Germain et al., 2019; Murray and Bremer, 1996; Sanyal and Harinarayanan, 2020). Considering the *gppA* and *spoT* genes as internal controls of the screen, we further focused our work on *nirD.*

**Figure 1—figure supplement 1.**
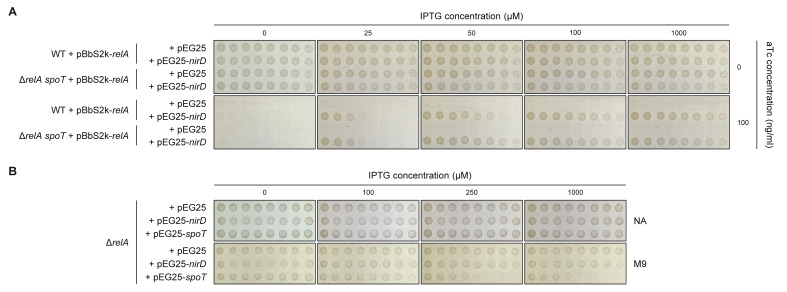
NirD can inhibit RelA-dependent (p)ppGpp synthesis. (**A**) Growth assay of the *E. coli* MG1655 wild-type strain and its isogenic Δ*relA spoT* mutant co-transformed with the plasmid derivatives pBbS2k-*relA* (sd4) and pEG25-*nirD* inducible by anhydrotetracycline (aTc) and isopropyl β-D-1-thiogalactopyranoside (IPTG), respectively. Serial dilutions of stationary-phase cultures were spotted on NA plates containing the indicated concentrations of inducers. The results are representative of three independent experiments. (**B**) Growth assay of the Δ*relA* mutant carrying pEG25-*nirD* or pEG25-*spoT*. Serial dilutions of stationary-phase cultures were spotted on M9-glucose minimal medium plates containing the indicated concentrations of IPTG. The results are representative of three independent experiments.

### NirD balances (p)ppGpp level produced by RelA

*nirD* encodes the small subunit of the cytoplasmic nitrite reductase (Harborne et al., 1992; Peakman et al., 1990a) and has never been linked to the (p)ppGpp metabolism. We first confirmed the result obtained with the high-copy-number vector pCA24N-*nirD* from the ASKA library by re-cloning the coding region of *nirD* (untagged) in a more suitable physiological plasmid harboring a tight IPTG-inducible P_T5-lac_ promoter (pEG25). Indeed, as shown in Figure 1B, NirD can robustly overcome the toxicity associated to (p)ppGpp over-accumulation mediated by RelA. To further assess the role of NirD in the stringent response, we monitored (p)ppGpp levels in vivo after consecutive inductions of RelA and NirD in a double expression system assay. As shown in Figure 1C, D, induction of RelA is associated with a massive accumulation of (p)ppGpp, which is sharply stopped and then decreased upon NirD induction.

The rapid (p)ppGpp decay observed following NirD induction raises the possibility that NirD may trigger SpoT-dependent (p)ppGpp hydrolysis. However, NirD still suppresses toxicity associated to RelA overproduction when the double expression assay experiment is performed in a Δ*relA spoT* background (Figure 1—figure supplement 1A). These results show that the hydrolase activity of SpoT is not involved in the reduced (p)ppGpp accumulation observed upon NirD induction. Importantly, overexpression of *nirD* in a Δ*relA* strain failed to phenocopy the well-known amino acid auxotrophic characteristic of a ppGpp^0^ strain (Δ*relA spoT* mutant) (Xiao et al., 1991), strongly arguing that NirD does not act as a small alarmone hydrolase (Figure 1—figure supplement 1B). Taken together, our results show that NirD impairs RelA-dependent (p)ppGpp synthesis in *E. coli*.

### NirD can prevent the stringent response in *E. coli*

**Figure 2.**
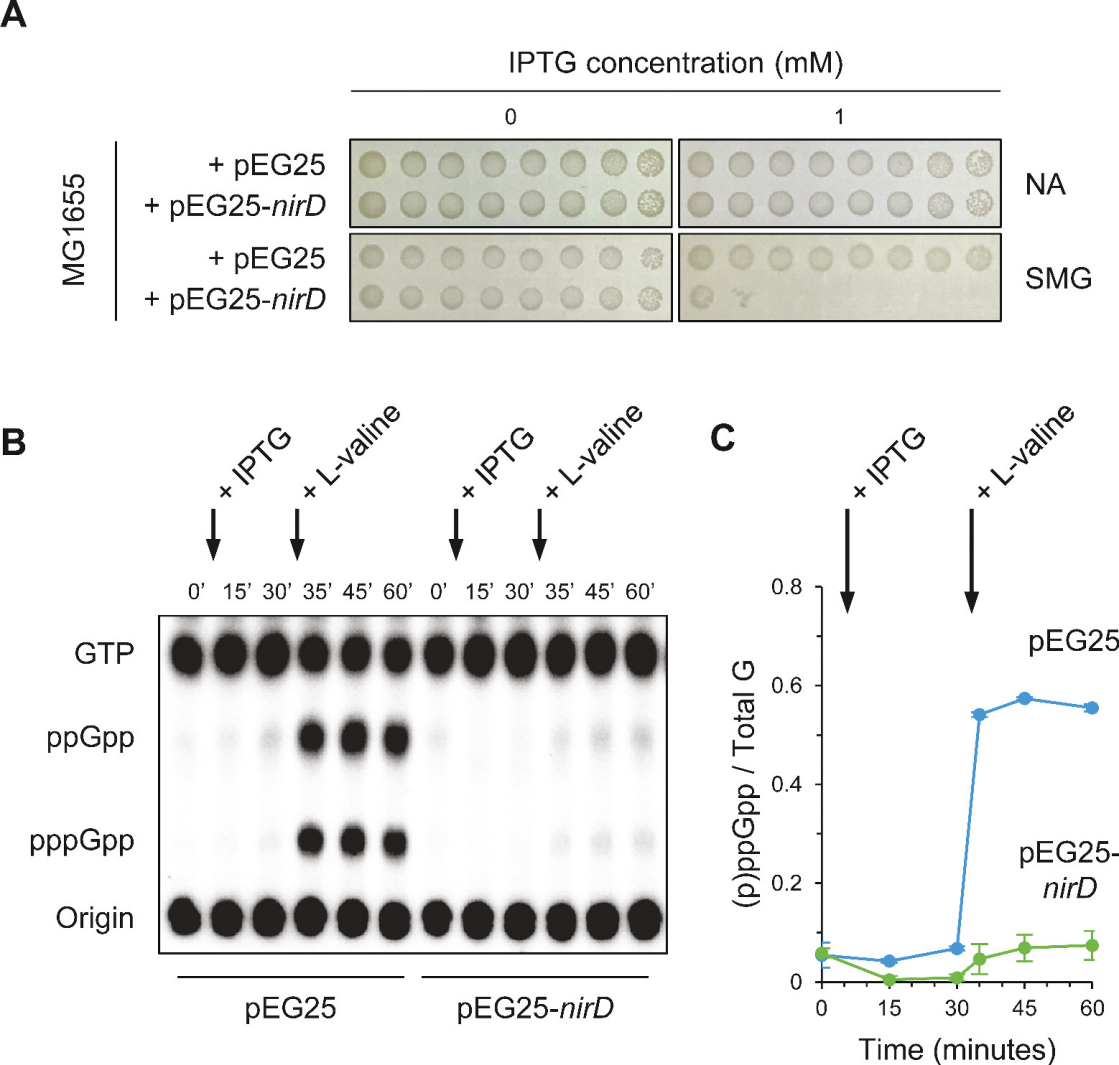
NirD inhibits accumulation of (p)ppGpp upon amino acid starvation. (**A**) NirD inhibits cell growth on M9-glucose minimal medium supplemented with serine, methionine, and glycine (SMG). Stationary-phase cultures of wild-type *E. coli* MG1655 strain harboring pEG25-*nirD* or pEG25-*spoT* were spotted on NA and SMG plates containing the indicated concentrations of isopropyl β-D-1-thiogalactopyranoside (IPTG). The results are representative of three independent experiments. (**B, C**) Exponentially growing cells of wild-type *E. coli* MG1655 harboring pEG25 or pEG25-*nirD* were challenged for amino acid starvation by addition of 500 µg/mL of L-valine. *nirD* is induced by the addition of 1 mM of IPTG. Nucleotides were extracted and separated by thin layer chromatography. The autoradiogram (**B**) is representative of three independent experiments, and the curves of the relative levels of (p)ppGpp (**C**) are represented as the means of the three independent experiments, the error bars depicting the SDs. Figure 2—source data 1.Raw autoradiogram. Figure 2—source data 2.Quantification of (p)ppGpp.The amount of (p)ppGpp was normalized to total amount of G nucleotides observed in each sample. Total G is the sum of GTP and (p)ppGpp detected. The source data are provided for the relative levels of (p)ppGpp in Figure 2C, which are represented as the means and SDs of three independent experiments.

Since NirD can efficiently overcome toxicity associated to artificial level of (p)ppGpp when RelA is overproduced (Figure 1B), we next address whether similar effect is observed during physiological condition known to activate RelA. In natural environment, RelA synthesizes the alarmones in response to amino acid deprivation. Indeed during amino acid starvation, deacylated tRNAs accumulate and RelA interacts with uncharged tRNA at the vacant ribosomal A-site causing the activation of its (p)ppGpp synthetic activity (Arenz et al., 2016; Brown et al., 2016; Loveland et al., 2016; Winther et al., 2018). RelA activity is therefore required to survive under amino acid starvation, and as a result a Δ*relA* mutant is not able to grow in the presence of 1 mM serine, methionine, and glycine (SMG), which induces isoleucine starvation (Uzan and Danchin, 1978). Interestingly, we observed that overexpression of *nirD* in wild-type (WT) strain phenocopies the growth defect of a Δ*relA* mutant on SMG plate (Figure 2A), suggesting that *nirD* also prevents (p)ppGpp accumulation from endogenous RelA under these conditions. To further address this possibility, we monitored the dynamics of (p)ppGpp levels under amino acid starvation upon the addition of L-valine (which also induces isoleucine starvation). As shown in Figure 2B, C, and as previously observed (p)ppGpp rapidly increases in WT strain and reaches maximum levels within 5 min (Cashel and Gallant, 1969). Remarkably, when NirD is induced prior to amino acid starvation, no significant accumulation of (p)ppGpp is detected (Figure 2B, C). Taken together, our results show that once induced NirD abolishes the stringent response in *E. coli.*

### NirD functionally interacts with RelA in vivo

**Figure 3.**
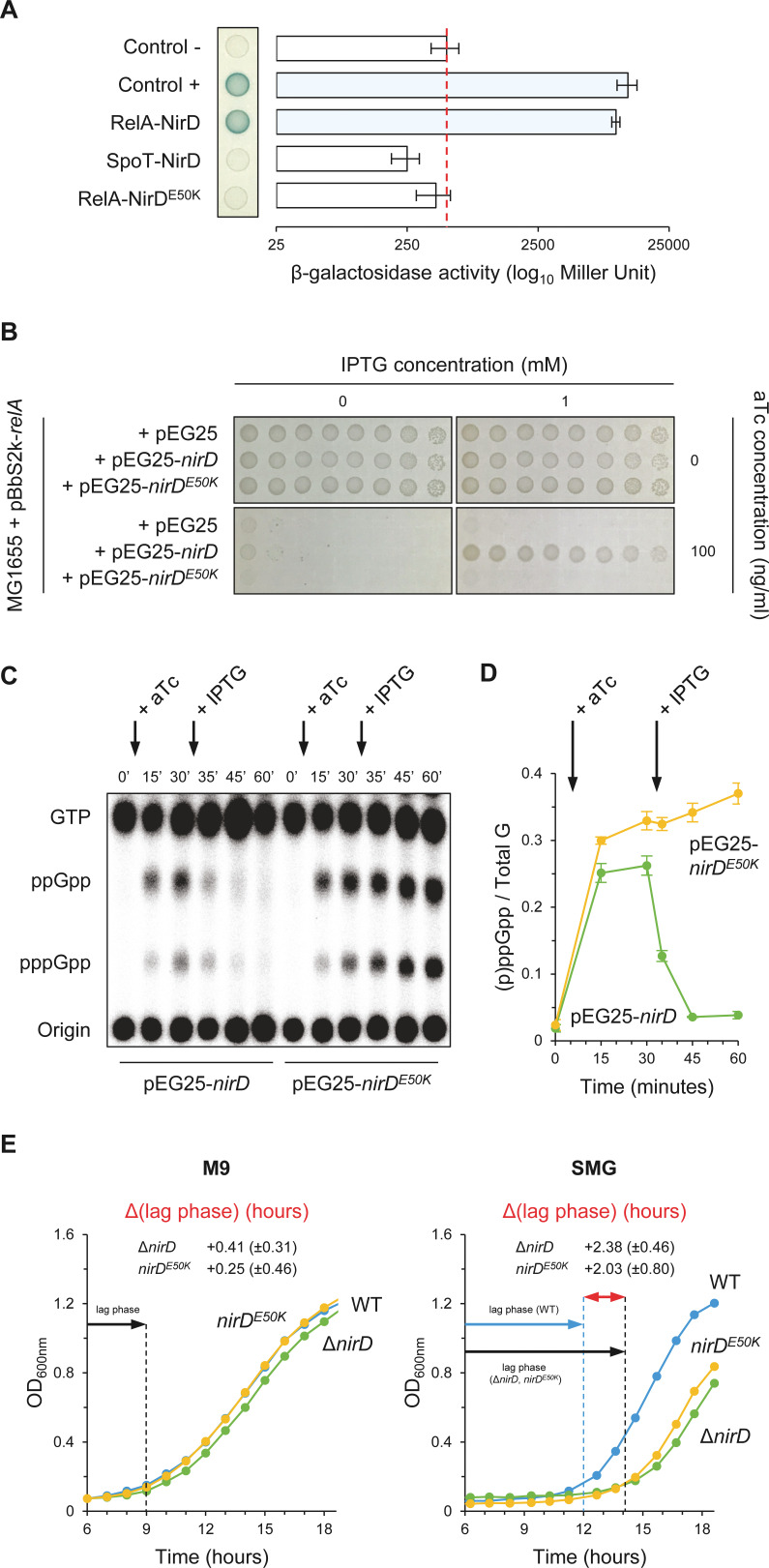
NirD can inhibit RelA-dependent accumulation through physical interaction in vivo. (**A**) Bacterial two-hybrid assay using *E. coli* BTH101 cells co-transformed with plasmid derivatives pKT25-*relA* or pKT25-*spoT* and pUT18C-*nirD* or pUT18C-*nirD^E50K^*. Stationary-phase cultures were spotted on NA plates containing X-Gal as a blue color reporter for positive interaction. The bars showing β-galactosidase activity are represented as the means of three independent experiments, and the error bars depict the SDs. (**B**) Growth assay of *E. coli* MG1655 cells co-transformed with the plasmid derivatives pBbS2k-*relA* and pEG25-*nirD* or pEG25-*nirD^E50K^* inducible by anhydrotetracycline (aTc) and isopropyl β-D-1-thiogalactopyranoside (IPTG), respectively. Serial dilutions of stationary-phase cultures were spotted on NA plates containing the indicated concentrations of inducers. The results are representative of three independent experiments. (**C, D**) In vivo (p)ppGpp level dynamic in *E. coli* MG1655 cells carrying pBbS2k-*relA* and pEG25-*nirD* or pEG25-*nirD^E50K^* after successive inductions of expression of the *relA* and *nirD* or *nirD^E50K^* genes using 100 ng/mL aTc and 1 mM IPTG, respectively. Nucleotides extracted from samples collected at the indicated times were separated by thin layer chromatography. The autoradiogram (**C**) is representative of three independent experiments, and the curves of the relative levels of (p)ppGpp (**D**) are represented as the means of the three independent experiments, the error bars depicting the SDs. (**E**) Growth curves of wild-type (WT) cells, Δ*nirD,* and *nirD^E50K^* mutants under anaerobiosis in M9-glucose minimal medium without amino acids (left panel) or supplemented with 1 mM serine, methionine, and glycine (SMG) (right panel) (see Materials and methods). The growth curves are representative of at least three independent experiments, and the Δ(lag phase) are represented as the means (± SD) of the independent experiments. Figure 3—source data 1.Determination of β-galactosidase activity.The relative β-galactosidase activity was calculated as follows:$1000\times \frac{Slope(\frac{{\Delta OD}_{420nm}}{\Delta t})}{V\times {OD}_{600nm}}$. Slope is estimated by linear regression, and V is the volume of culture used (in mL). The source data are provided for the bar chart showing β-galactosidase activity in Figure 3A, which is represented as the means and SDs of three independent experiments. Figure 3—source data 2.Raw autoradiogram. Figure 3—source data 3.Quantification of (p)ppGpp.The amount of (p)ppGpp was normalized to total amount of G nucleotides observed in each sample. Total G is the sum of GTP and (p)ppGpp detected. The source data are provided for the relative levels of (p)ppGpp in Figure 3D, which are represented as the means and SDs of three independent experiments. Figure 3—source data 4.Determination of Δ(lag phase).The Δ(lag phase) corresponds to the time that separates the growth recovery of the wild-type (WT) (lag phase [WT]) from that of the Δ*nirD* or *nirD^E50K^* (lag phase [Δ*nirD*, *nirD^E50K^*]). For each strain, the duration of the lag phase was calculated for an OD_600nm_ corresponding to the entry into the exponential growth phase. The equation for this calculation was estimated by linear regression on the log phase of each growth curve. The source data are provided for the Δ(lag phase) in Figure 3E, which are represented as the means and SDs of at least three experiments.

Regulation of RSH activities through protein–protein interactions has been previously reported (Battesti and Bouveret, 2006; Germain et al., 2019; Hahn et al., 2015; Karstens et al., 2014; Krüger et al., 2020; Lee et al., 2018; Peterson et al., 2020; Raskin et al., 2007; Ronneau et al., 2016; Ronneau et al., 2019; Wout et al., 2004). We therefore tested whether NirD is able to interact with RelA in vivo using a bacterial two-hybrid (BTH) assay (Karimova et al., 1998). For that purpose, the complementary T18 and T25 domains of *Bordetella pertussis* adenylate cyclase (CyA) were fused to the N-terminus of NirD and RelA proteins using the two compatible vectors pUT18C and pKT25, respectively. When the *E. coli* BTH101 strain (*cya* deficient) is co-transformed with plasmids harboring T18 and T25 fusions, NirD exhibited a strong interaction in vivo with RelA (Figure 3A). However, despite the strong sequence homologies between the paralogous RelA and SpoT proteins (Metzger et al., 1989), no physical interaction is observed between SpoT and NirD in vivo by BTH assay (Figure 3A). This result therefore supports a strong specificity of the interaction between NirD and RelA.

**Figure 3—figure supplement 1.**
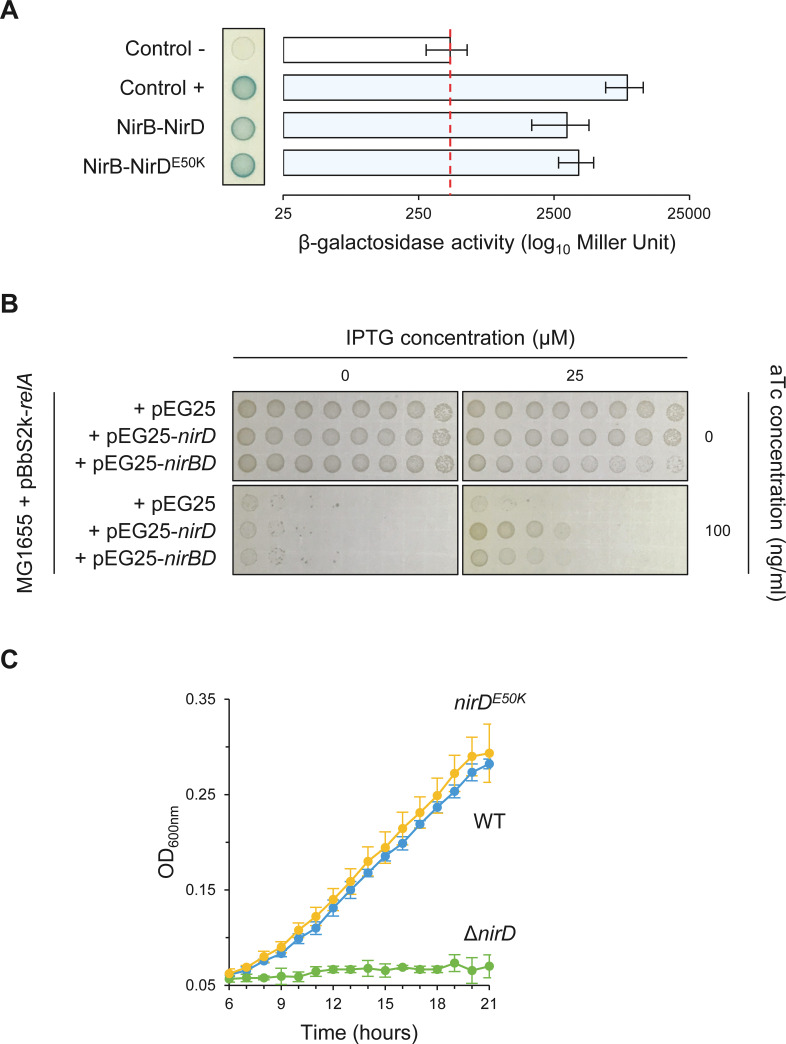
NirD^E50K^ functionally interacts with NirB. (**A**) Bacterial two-hybrid assay using *E. coli* BTH101 cells co-transformed with plasmid derivatives pKT25-*nirB* and pUT18C-*nirD* or pUT18C-*nirD^E50K^*. Stationary-phase cultures were spotted on NA plates containing X-Gal as a blue color reporter for positive interaction. The bars showing β-galactosidase activity are represented as the means of three independent experiments, and the error bars depict the SDs. (**B**) Growth assay of *E. coli* MG1655 cells co-transformed with the plasmid derivatives pBbS2k-*relA* and pEG25-*nirD* or pEG25-*nirBD* inducible by anhydrotetracycline (aTc) and isopropyl β-D-1-thiogalactopyranoside (IPTG), respectively. Serial dilutions of stationary-phase cultures were spotted on NA plates containing the indicated concentrations of inducers. The results are representative of three independent experiments. (**C**) Growth curves of wild-type (WT) cells, Δ*nirD,* and *nirD^E50K^* mutants under anaerobiosis in defined glycerol minimal medium supplemented with nitrate as the sole electron acceptor and as the sole nitrogen source (see Materials and methods). Under nitrate respiration, nitrate is converted to nitrite, which is subsequently converted to ammonium (NH_4_) by the cytosolic NADH-dependent nitrite reductase NirBD. Bacteria can then utilize NH_4_ as nitrogen source. Therefore, an active NADH-dependent nitrite reductase activity is required for anaerobic growth when nitrate is the sole nitrogen source (Wang et al., 2019b). Figure 3—figure supplement 1—source data 1.Determination of β-galactosidase activity.The relative β-galactosidase activity was calculated as follows:$1000\times \frac{Slope(\frac{{\Delta OD}_{420nm}}{\Delta t})}{V\times {OD}_{600nm}}$. Slope is estimated by linear regression, and V is the volume of culture used (in mL). The source data are provided for the bar chart showing β-galactosidase activity in Figure 3—figure supplement 1A, which is represented as the means and SDs of three independent experiments. Figure 3—figure supplement 1—source data 2.Raw data for growth curves.The source data are provided for the growth curves in Figure 3—figure supplement 1C, which are represented as the means and SDs of three independent experiments.

To further assess the significance of the NirD-RelA interaction in vivo and its role in (p)ppGpp homeostasis, we randomly mutagenized *nirD* and searched for defective mutants unable to interact with RelA. One of such mutants that had charge reversal amino acid substitution, glutamate 50 to lysine (E50K), displayed compromised interaction with RelA (Figure 3A). Importantly, we confirm that both T18-NirD and T18-NirD^E50K^ recombinant proteins were correctly expressed and folded as shown by their ability to strongly interact with the catalytic subunit of the nitrite reductase NirB (Figure 3—figure supplement 1) (Harborne et al., 1992). Importantly and on the contrary to the results obtained with the WT copy of *nirD*, we observed that overexpression of *nirD^E50K^* is not able to suppress toxicity associated to RelA overproduction on plate (Figure 3B). This result predicts that breaking the interaction with RelA abolishes the inhibitory effect of NirD on (p)ppGpp accumulation. Indeed, as shown in Figure 3C, D, *nirD^E50K^* failed to stop the massive accumulation of (p)ppGpp observed in vivo after RelA overproduction as compared to the fast decay of (p)ppGpp observed when the WT copy of *nirD* is expressed (Figure 3C and D).

*nirD* is a second gene of a four-gene operon (*nirBDC-cysG*) strongly induced under anaerobic growth (Harborne et al., 1992; Peakman et al., 1990b). Interestingly, we observed that Δ*nirD* mutant presents an extended growth recovery in anaerobic SMG liquid medium consistent with a toxic over-accumulation of (p)ppGpp in this strain (Figure 3E). Indeed, a chromosomal encoded *nirD^E50K^* mutant, which is functional for the nitrite reductase activity (Figure 3—figure supplement 1C) but failed to interact with RelA, displays the same growth defect, suggesting that the NirD-RelA interaction is important to adjust proper (p)ppGpp level under this specific growth condition. Taken together, our results show that a specific functional interaction between RelA and NirD can lower RelA-dependent accumulation of (p)ppGpp in vivo.

### NirD targets the catalytic N-terminal region of RelA

**Figure 4.**
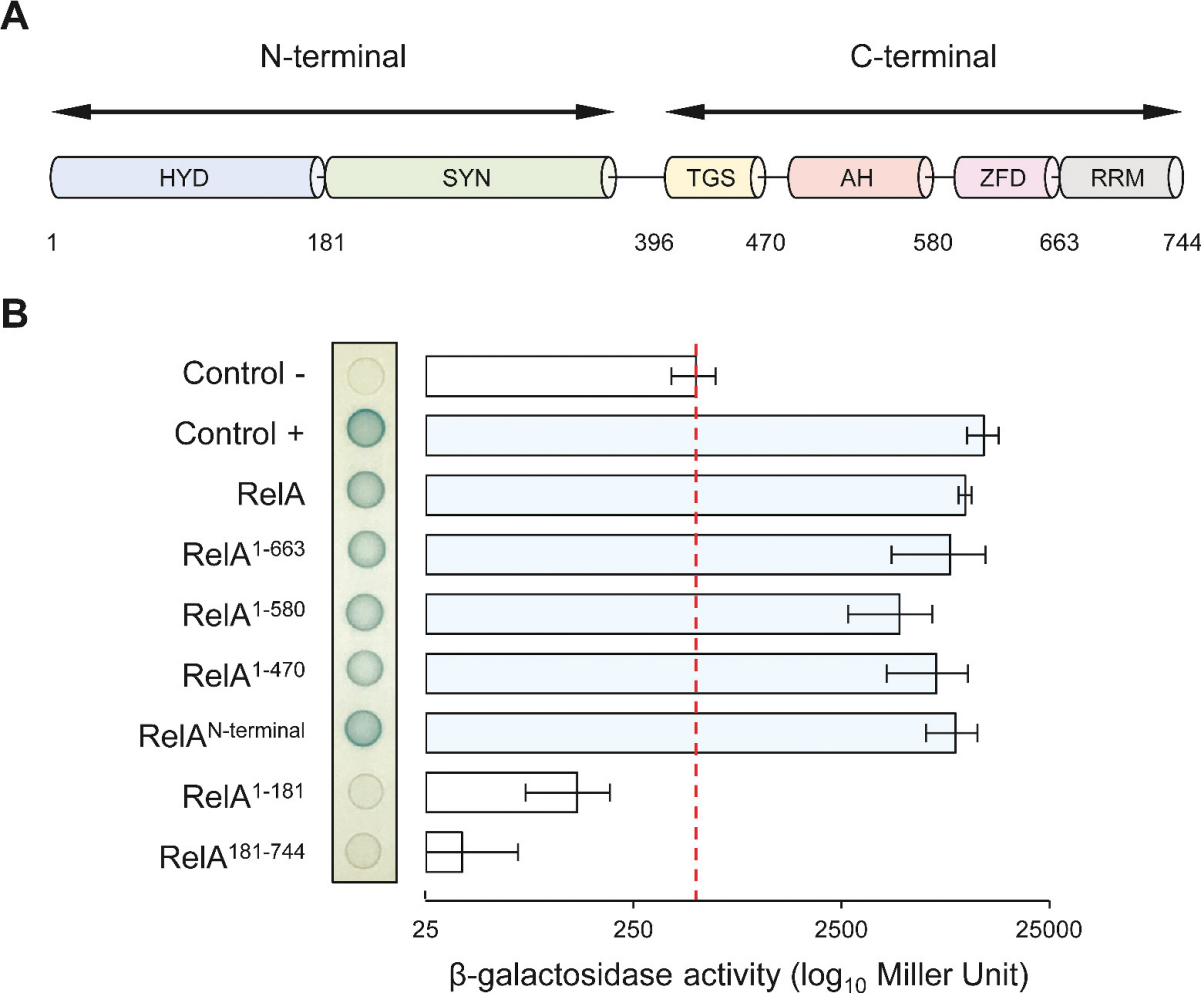
NirD can interact with the catalytic N-terminal region of RelA. (**A**) Schematic representation of RelA and its protein domains. (**B**) Bacterial two-hybrid assay using *E. coli* BTH101 cells co-transformed with plasmid derivatives pUT18C-*nirD* and pKT25 with the full-length or truncated *relA* gene as indicated. Stationary-phase cultures were spotted on NA plates containing X-Gal as a blue color reporter for positive interaction. The bars showing β-galactosidase activity are represented as the means of three independent experiments, and the error bars depict the SDs. Figure 4—source data 1.Determination of β-galactosidase activity.The relative β-galactosidase activity was calculated as follows:$1000\times \frac{Slope(\frac{{\Delta OD}_{420nm}}{\Delta t})}{V\times {OD}_{600nm}}$. Slope is estimated by linear regression, and V is the volume of culture used (in mL). The source data are provided for the bar chart showing β-galactosidase activity in Figure 4B, which is represented as the means and SDs of three independent experiments.

RelA consists of several protein domains that have been encompassed in two functional regions, the catalytic NTD and the regulatory CTD. The N-terminal region includes the enzymatically inactive (p)ppGpp hydrolase domain (HYD) as well as the functional (p)ppGpp synthetase domain (SYN), while the CTD comprises the Thr-tRNA synthetase, GTPase and SpoT domain (TGS), the alpha helical domain (AH), the zinc finger domain (ZFD), and finally the RNA recognition motif domain (RRM) (Figure 4A) (Brown et al., 2016; Loveland et al., 2016). To determine which of these domains are required for the interaction with NirD, we assayed the interaction between NirD and several truncated RelA proteins using the BTH assay.

**Figure 4—figure supplement 1.**
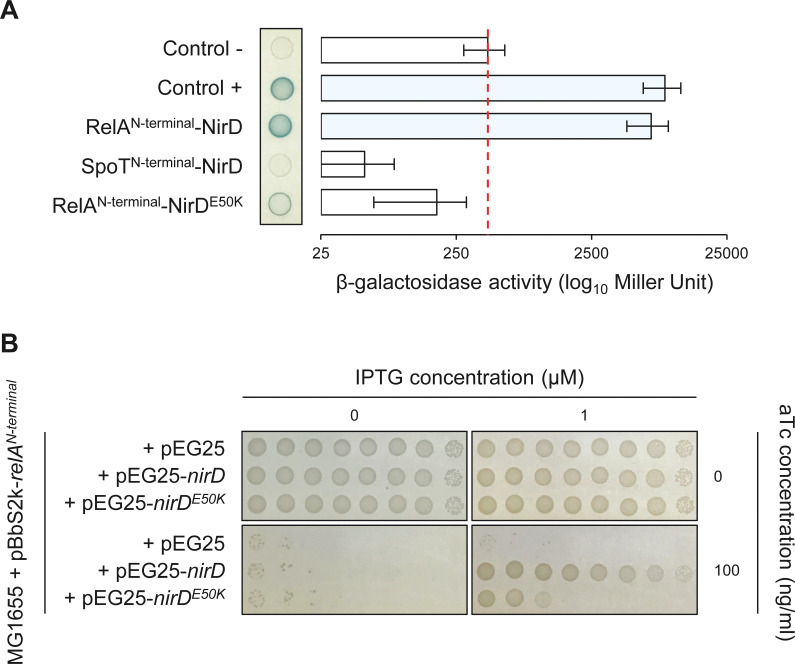
The RelA^N-terminal^-NirD interaction appears specific. (**A**) Bacterial two-hybrid assay using *E. coli* BTH101 cells co-transformed with plasmid derivatives pKT25-*relA^N-terminal^* or pKT25-*spoT^N-terminal^* and pUT18C-*nirD* or pUT18C-*nirD^E50K^*. Stationary-phase cultures were spotted on NA plates containing X-Gal as a blue color reporter for positive interaction. The bars showing β-galactosidase activity are represented as the means of three independent experiments, and the error bars depict the SDs. (**B**) Growth assay of *E. coli* MG1655 cells co-transformed with the plasmid derivatives pBbS2k-*relA^N-terminal^* and pEG25-*nirD* or pEG25-*nirD^E50K^* inducible by anhydrotetracycline (aTc) and isopropyl β-D-1-thiogalactopyranoside (IPTG), respectively. Serial dilutions of stationary-phase cultures were spotted on NA plates containing the indicated concentrations of inducers. The results are representative of three independent experiments. Figure 4—figure supplement 1—source data 1.Determination of β-galactosidase activity.The relative β-galactosidase activity was calculated as follows:$1000\times \frac{Slope(\frac{{\Delta OD}_{420nm}}{\Delta t})}{V\times {OD}_{600nm}}$. Slope is estimated by linear regression, and V is the volume of culture used (in mL). The source data are provided for the bar chart showing β-galactosidase activity in Figure 4—figure supplement 1A, which is represented as the means and SDs of three independent experiments.

Truncated RelA proteins were fused to the T25 domain of the *B. pertussis* adenylate cyclase and transformed together with NirD fused to the T18 domain in a *cya*-deficient *E. coli* strain (BTH101). BTH analysis revealed that NirD interacts with the RelA^N-terminal^ fusion comprising the catalytic domains (Figure 4B). Moreover, careful quantification of β-galactosidase activity revealed that none of the four domains encompassing the CTD of RelA is required for this interaction in vivo. However, truncated RelA fusion lacking either the synthetase domain (RelA^1-181^) or the ‘pseudo’ hydrolase domain (RelA^181-744^) failed to interact in vivo with NirD. Therefore, the catalytic domains of RelA seem necessary and sufficient to interact with NirD in vivo. The specificity of the interaction between NirD and RelA was further reinforced by the absence of interaction observed between NirD and SpoT^N-terminal^ as well as NirD^E50K^ and RelA^N-terminal^ (Figure 4—figure supplement 1A). Interestingly, the C-terminal region of RelA is proposed to play a key role in the autoregulation of the enzymatic activity, and as a result the RelA^N-terminal^ truncated protein is constitutively active (Schreiber et al., 1991). Given that NirD interacts with RelA^N-terminal^, we therefore tested the intriguing possibility that NirD may also be able to inhibit this dysregulated variant. Remarkably, as shown in Figure 4—figure supplement 1B, we observed that NirD but not NirD^E50K^ inhibits the toxicity associated with (p)ppGpp over-accumulation mediated by the constitutively active RelA^N-terminal^. Taken together, our results show that NirD can inhibit RelA activity by interacting with its catalytic N-terminal region in vivo.

### NirD directly binds RelA to inhibit the rate of (p)ppGpp synthesis in vitro

To confirm the in vivo interaction of NirD at the catalytic N-terminal region of RelA, we used different complementary in vitro methods. For that purpose, the RelA catalytic domains (RelA^N-terminal^), NirD, and NirD^E50K^ were individually produced and purified by two consecutive chromatographies, affinity and size-exclusion (Figure 5—figure supplement 1A–C). We first performed size-exclusion chromatography (SEC) experiment separating RelA^N-terminal^ and NirD alone or pre-mixed. As shown in Figure 5—figure supplement 1D, co-elution resulted in an earlier eluting peak, suggesting the formation of a stable complex. Indeed, SDS-PAGE analysis confirmed that the two proteins were co-eluted from the column (Figure 5—figure supplement 1E).

**Figure 5.**
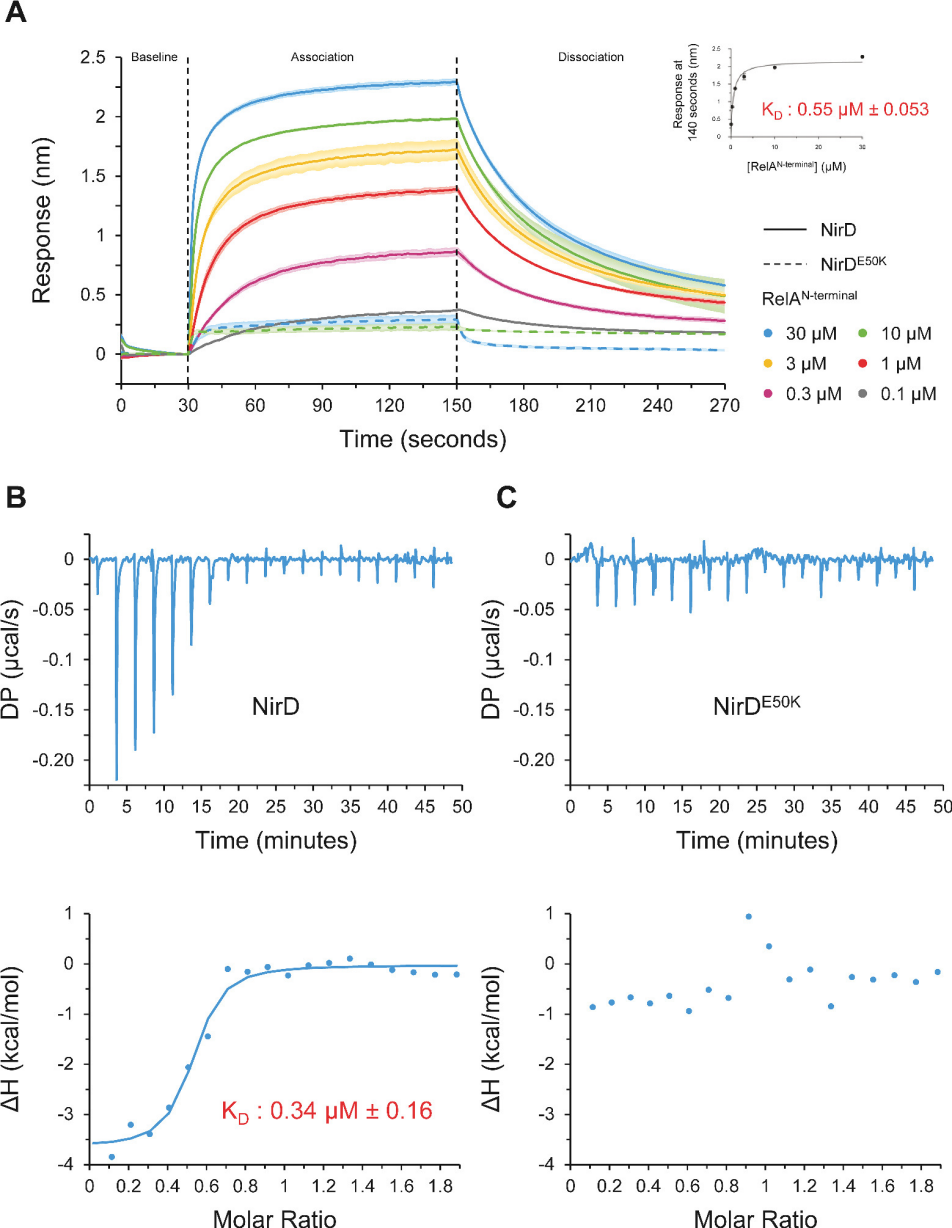
NirD directly interact with the catalytic N-terminal region of RelA. (**A**) Biolayer interferometric assay of RelA^N-terminal^ on NirD or NirD^E50K^. Biotinylated NirD or NirD^E50K^ were immobilized on streptavidin biosensors and probed with RelA^N-terminal^ at concentrations ranging from 0.1 to 30 µM. The curves are represented as the means of the subtracted reference binding responses during association and dissociation from three experiments, the error bars depicting the SDs. The inset curve shows the specific biolayer interferometry (BLI) response (nm) 10 s before the end of association as a function of RelA^N-terminal^ concentration. Data are represented as the means of the three experiments, the error bars depicting the SDs. (**B, C**) Isothermal titration calorimetry profiles corresponding to the binding of RelA^N-terminal ^30–300 µM NirD (**B**) or 300 µM NirD^E50K^ (**C**) at 25°C. The upper panels show raw data for titration of NirD or NirD^E50K^ with RelA^N-terminal^, and the lower panels show the integrated heats of binding obtained from the raw data. The data were fitted using a ‘One Set of Sites’ model in the PEAQ-ITC Analysis Software. Figure 5—source data 1.Raw data for biolayer interferometry (BLI) experiments.The source data are provided for the BLI experiments in Figure 5A, which are represented as the means and SDs of three independent experiments. Figure 5—source data 2.Raw data for isothermal titration calorimetry experiment run with NirD. Figure 5—source data 3.Raw data for isothermal titration calorimetry experiment run with NirD^E50K^.

To further characterize the NirD-RelA^N-terminal^ interaction, we then used biolayer interferometry (BLI), an in vitro approach for measuring biomolecular interactions. Biotinylated NirD was immobilized on streptavidin biosensors as ligand, and RelA^N-terminal^ was used as the analyte. By adding RelA at concentrations ranging from 0.1 to 30 µM, a dose-response association is recorded and decreased during the dissociation step corresponding to the washing of the sensor (Figure 5A), showing that NirD directly interact with the catalytic N-terminal region of RelA. Moreover, by plotting the maximum binding response against the corresponding concentrations of RelA, we calculated a dissociation constant (K_D_) of 0.55 µM (± 0.053 µM) (Figure 5A). In addition, and in agreement with the BTH assays (Figure 4—figure supplement 1A), RelA^N-terminal^ did not interact with the NirD^E50K^ variant in vitro (Figure 5A). Finally, to further determine the thermodynamic parameters of the NirD-RelA^N-terminal^ interaction, we performed isothermal titration calorimetry (ITC) experiment. The assay first confirmed the direct interaction between the two proteins with an estimated K_D_ of 0.34 µM (Figure 5B), very similar to that estimated by BLI (Figure 5A). This reaction is a favorable spontaneous exothermic reaction with a ΔH kcal/mol of −3.63 ± 0.208 and is enthalpy driven at 25°C. On the contrary and consistent with BLI experiments, no heat exchange was recorded upon titration of RelA^N-terminal^ by NirD^E50K^, further supporting the strong specificity of the interaction between NirD and RelA (Figure 5B). Collectively, these results show that NirD interacts directly and specifically with the catalytic domains of RelA in vitro.

**Figure 5—figure supplement 1.**
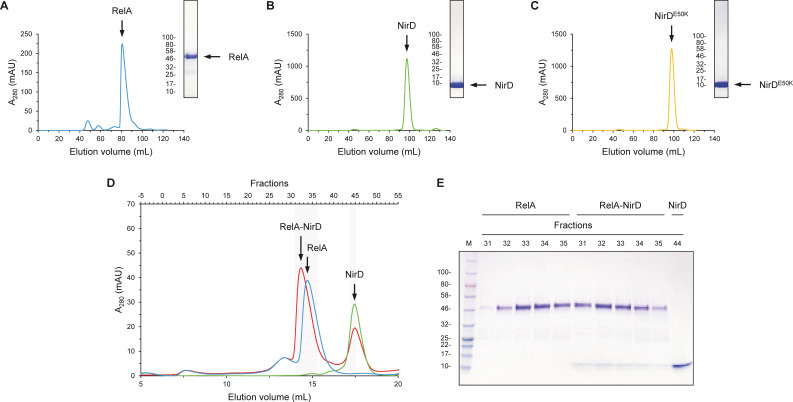
NirD can directly interact with the catalytic N-terminal region of RelA. (**A–C**) Purification of RelA^N-terminal^ (**A**), NirD (**B**), and NirD^E50K^ (**C**) using size-exclusion chromatography (SEC). The curves show the absorbance at 280 nm as a function of the elution volume. The SEC elution fractions indicated by arrows were analyzed by 12% SDS-PAGE. For SDS-PAGE, molecular weight markers are expressed in kDa. (**D, E**) SEC experiment separating RelA^N-terminal^ and NirD alone or pre-mixed. (**D**) The curves show the absorbance at 280 nm as a function of the elution volume. The SEC elution fractions highlighted were analyzed by 12% SDS-PAGE (**E**). For SDS-PAGE, molecular weight markers are expressed in kDa. Figure 5—figure supplement 1—source data 1.Raw data for purification of RelA^N-terminal^. Figure 5—figure supplement 1—source data 2.Raw SDS-PAGE for RelA^N-terminal^ purification. Figure 5—figure supplement 1—source data 3.Raw data for purification of NirD. Figure 5—figure supplement 1—source data 4.Raw SDS-PAGE for NirD purification. Figure 5—figure supplement 1—source data 5.Raw data for purification of NirD^E50K^. Figure 5—figure supplement 1—source data 6.Raw SDS-PAGE for NirD^E50K^ purification. Figure 5—figure supplement 1—source data 7.Raw data for size-exclusion chromatography experiment separating RelA^N-terminal^ and NirD alone or pre-mixed. Figure 5—figure supplement 1—source data 8.Raw SDS-PAGE for size-exclusion chromatography experiment.

**Figure 6.**
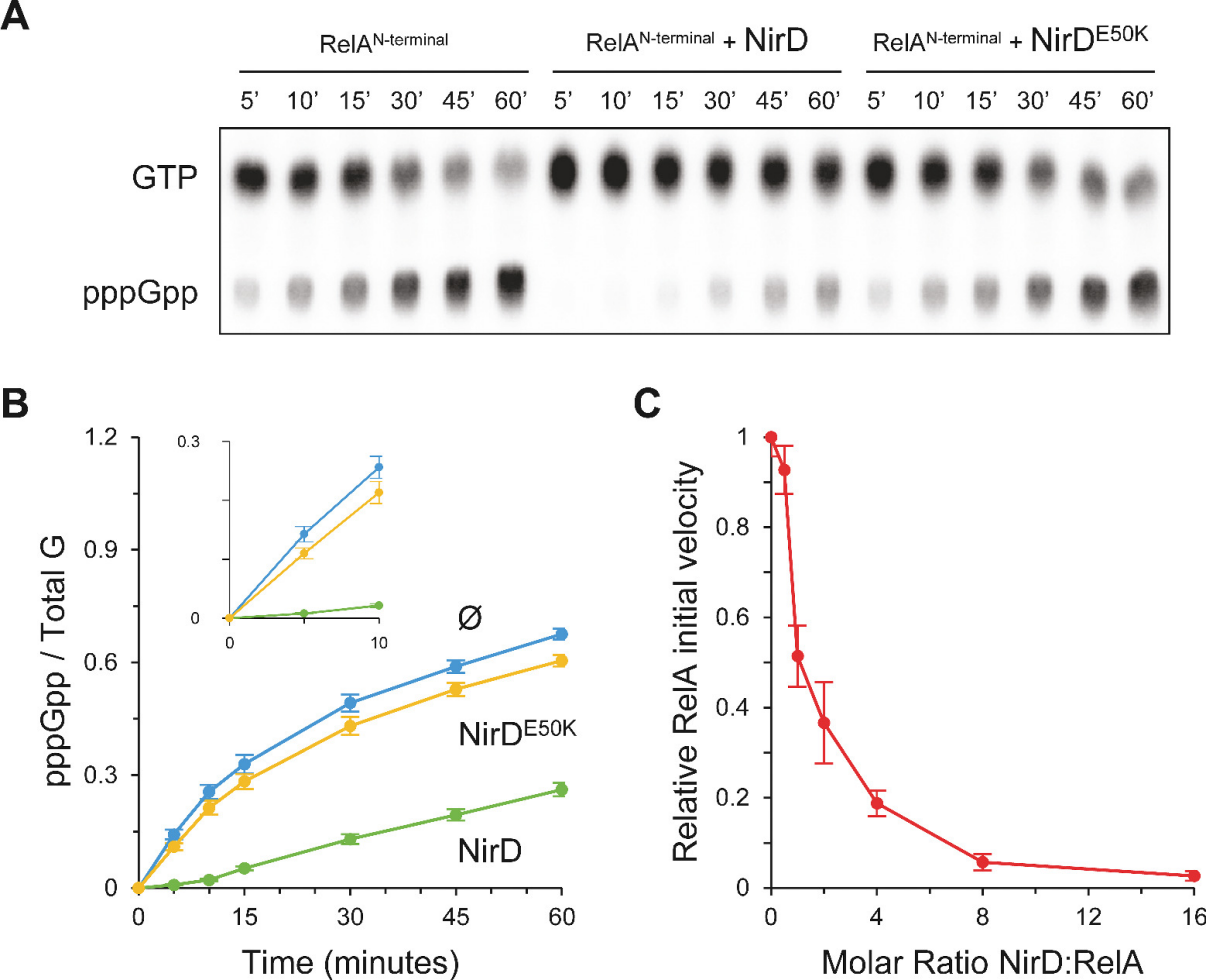
NirD inhibits RelA activity in vitro. (**A, B**) In vitro pppGpp formation by 1 µM RelA^N-terminal^ in the presence or absence of 4 µM NirD or NirD^E50K^. Samples collected at the indicated times were separated by thin layer chromatography (TLC). The autoradiogram (**A**) is representative of three experiments, and the curves of the relative levels of pppGpp (**B**) are represented as the means of the three experiments, the error bars depicting the SDs. (**C**) Relative initial velocity of RelA as a function of NirD:RelA molar ratio. The in vitro formation of pppGpp by 1 µM RelA^N-terminal^ was assayed in the presence or absence of NirD at concentrations ranging from 0.5 to 16 µM. Samples were collected every 30 s over a period of 150 s and separated by TLC to determine the relative initial velocity (estimated by linear regression using five data points). Results are represented as the means of three experiments, and the error bars depict the SDs. Figure 6—source data 1.Raw autoradiogram. Figure 6—source data 2.Quantification of pppGpp.The amount of pppGpp was normalized to total amount of G nucleotides observed in each sample. Total G is the sum of GTP and pppGpp detected. The source data are provided for the relative levels of pppGpp in Figure 6B, which are represented as the means and SDs of three independent experiments. Figure 6—source data 3.Determination of the relative initial velocity.The relative initial velocity was estimated on the linear part of the in vitro pppGpp formation by linear regression at the very beginning of the reaction. The source data are provided for the results in Figure 6C, which are represented as the means and SDs of three experiments.

Finally, these results are in line with our in vivo results showing that NirD inhibits accumulation of (p)ppGpp from the constitutively active RelA^N-terminal^ protein (Figure 4—figure supplement 1B) and therefore support a rather simple model where NirD would directly inhibit RelA synthetic activity through direct binding. To challenge this hypothesis, we performed in vitro pppGpp synthesis assay with purified proteins. We observed that RelA^N-terminal^ protein could efficiently synthetize [^32^P]-pppGpp, but the addition of NirD strongly affected the synthetase activity in vitro. Indeed, 80% of GTP are converted to pppGpp within 60 min by RelA, whereas only 30% are converted when NirD is added (Figure 6A, B). Importantly, addition of the NirD^E50K^ does not affect the RelA synthetic activity (Figure 6A, B). Finally, careful examination of the initial rates (calculated over the first 150 s) shows a concentration-dependent inhibition of RelA’s (p)ppGpp synthetic activity by NirD with a half-inhibition occurring at ∼1:1 molar ratio and a near complete inhibition for the highest tested concentration of NirD (1:16 molar ratio) (Figure 6C). Taken together, these results show that NirD can block pppGpp synthetase activity of RelA through a direct interaction with the catalytic domains.

## Discussion

*E. coli* contains two long RSH enzymes, RelA and SpoT, that synthesize the alarmones (p)ppGpp. While SpoT is a bifunctional enzyme, RelA is monofunctional with a degenerated hydrolase domain, making SpoT the primary source of hydrolysis (Xiao et al., 1991). Therefore, a fine-tune regulation of the intracellular (p)ppGpp synthetic and hydrolytic activities in bacteria is crucial to rapidly adjust (p)ppGpp level in response to environmental stress but also to prevent toxic consequences due to (p)ppGpp over-accumulation (Xiao et al., 1991). Over the past decade, several molecular mechanisms regulating the activity of (p)ppGpp synthetase and/or hydrolase have been characterized (Battesti and Bouveret, 2006; Germain et al., 2019; Hahn et al., 2015; Karstens et al., 2014; Krüger et al., 2020; Lee et al., 2018; Peterson et al., 2020; Raskin et al., 2007; Ronneau et al., 2016; Ronneau et al., 2019; Wout et al., 2004). In this study, we uncovered an unprecedented mode of regulation that can prevent accumulation of (p)ppGpp in *E. coli*. This regulation implicates physical interaction in vivo and in vitro between the catalytic domains of RelA and the protein NirD, the small subunit of the cytoplasmic NADH-dependent nitrite reductase complex NirBD (Harborne et al., 1992). We show that NirD can functionally and directly inhibit the RelA (p)ppGpp synthetic activity. RelA and SpoT are paralogous proteins; however, despite strong sequence homologies and similar domain architecture we were not able to notice physical interaction between NirD and SpoT (Figure 3A and Figure 4—figure supplement 1A), supporting a specific physiological role of NirD in inhibiting RelA synthetic activity. The absence of a functional link between NirD and SpoT is further reinforced by the observation that NirD does not seem to lower basal (p)ppGpp level produced by the weak (p)ppGpp synthetase activity encoded by SpoT in the absence of RelA (Figure 1—figure supplement 1B).

Stimulation of RelA synthetase activity in *E. coli* is driven by stalled ribosomes upon amino acid starvation (Haseltine and Block, 1973; Wendrich et al., 2002; Winther et al., 2018). In addition, NtrC-dependent transcription of *relA* takes an important role in (p)ppGpp accumulation (Brown et al., 2014). Therefore, the identification of a potent intracellular inhibitor of RelA (p)ppGpp synthetase represents an additional level of complexity in the regulation to the current stringent model mediated by RelA. Our results show that the NirD protein level can play an important role for adjusting intracellular (p)ppGpp levels. Indeed, ectopic induction of NirD is sufficient to prevent accumulation of (p)ppGpp mediated by RelA in the absence of nutritional stress (Figure 1C, D). Moreover, we show that once expressed, *nirD* can also totally abolish activation of the stringent response during amino acid starvation (Figure 2C, D). The estimated concentration of RelA is in the range of 100–400 nM in *E. coli* (Li et al., 2014; Pedersen and Kjeldgaard, 1977). Importantly, we found in vitro that the NirD-RelA affinity is characterized by a K_D_ in this concentration range (300–550 nM) (Figure 5A, B). Therefore, at concentrations above the K_D_, NirD would interact with RelA to catalytically inhibit its activity. *nirD* is a second gene of a four-gene operon (*nirBDC-cysG*) expressed under anaerobic growth (Harborne et al., 1992; Peakman et al., 1990b) and maximally induced in the presence of glucose and nitrate (Tyson et al., 1997; Wang and Gunsalus, 2000). Under these conditions, NirD is predicted to reach µM range (Khlebodarova et al., 2016) and regulation of RelA by NirD would therefore become physiologically relevant. Importantly, while NirB and NirD can form a stable complex needed for the NADH-dependent nitrite reductase activity, we observed that coexpression of *nirB* and *nirD* can still counteract RelA-dependent (p)ppGpp accumulation (Figure 3—figure supplement 1B). The cytoplasmic nitrite reductase NirBD has emerged as a key regulator of the nitric oxide (NO) level during nitrate respiration (Bulot et al., 2019). Indeed, by reducing nitrite generated during nitrate reduction, NirBD would limit the level of toxic nitrite and thus its further reduction to NO (Bulot et al., 2019). In addition, our results present evidence that NirD can also promote bacterial fitness by adjusting (p)ppGpp levels in response to amino acid starvation under anaerobic glucose fermentation, a condition where the nitrite reductase is expressed but where the substrate is missing (Figure 3E). This regulation is dependent on the RelA-NirD interaction since NirD^E50K^, which is not affected for the nitrite reductase activity (Figure 3—figure supplement 1C) but failed to interact with RelA (Figure 3A), does not enhance bacterial fitness in response to amino acid starvation under anaerobic glucose fermentation (Figure 3E). Therefore, our results suggest that NirD can link (p)ppGpp homeostasis to anaerobic metabolism in *E. coli*. Additional analyses are currently underway to further investigate the physiological roles of the RelA-NirD interaction in *E. coli*.

The CTD of RSH enzymes is assumed to play key roles in sensing nutrient starvation and adjusting the enzymatic state of the NTD. In that sense, the RelA structures on stalled ribosomes emphasized the role of CTD in sensing nutrient availability. Once bound to the ribosome, RelA adopts an open extended conformation, which is thought to release the autoinhibitory effect of the CTD on the NTD, therefore promoting (p)ppGpp synthesis (Arenz et al., 2016; Brown et al., 2016; Loveland et al., 2016). Interestingly, we observed that the NTD region of RelA is necessary and sufficient for NirD binding (Figure 4B and Figure 5). Moreover, our in vivo and in vitro results convincingly show that NirD can inhibit the (p)ppGpp synthesis from the constitutively active truncated RelA^N-terminal^ (Figure 6 and Figure 4—figure supplement 1B), supporting the idea of a ribosome-independent inhibition of the stringent response. Therefore, rather than preventing activation of RelA our results support a simple model of regulation in which NirD directly interacts with the catalytic domains of RelA to inhibit the synthetic activity. Such inhibition might be mediated by directly introducing structural changes in the catalytic domains. Alternatively, NirD may interact with RelA to avoid nucleotide binding by masking the nucleotide-binding site. Structural and functional analyses are currently under investigation to test whether direct binding of NirD inhibits RelA activity.

Finally, (p)ppGpp has emerged as an important regulator of not only the bacterial stress response but also of many aspects of bacterial physiology including virulence, immune evasion, and antibiotic tolerance. Therefore, strategies to inhibit RelA activity and the subsequent production of (p)ppGpp represent an attractive approach for the success of antimicrobial therapy. Currently, there are only a limited number of synthetic inhibitors known to target RelA, mainly based on the design of (p)ppGpp analogues (Wexselblatt et al., 2012; Wexselblatt et al., 2013). Hence, our identification that NirD constitutes a natural potent inhibitor of RelA in vivo and in vitro could represent an important resource for the future design of new drug compounds. Importantly, it is also likely to be a fascinating molecular tool to further tackle the functional and structural determinant of the stringent response.

## Materials and methods

### Experimental model and subject details

The *E. coli* strains and plasmids used in this study are listed in Appendix 1—key resources table. Cells were grown in lysogeny broth (LB) broth (Miller, 1972) with agitation or on NA (Thermo Fisher Scientific, MA). For cells containing plasmid(s), the media were appropriately supplemented with the antibiotic(s) kanamycin (25 µg/mL), chloramphenicol (50 µg/mL), and/or ampicillin (50 µg/mL).

### Plasmids construction

The plasmid derivatives were constructed by cloning into a plasmid a gene amplified by PCR from template DNA (chromosomal or plasmid) using the primers listed in Appendix 1—key resources table. The restriction enzymes as well as Shine-Dalgarno sequence for the pEG25 and pBbS2k plasmids are also indicated (Appendix 1—key resources table).

### Multicopy suppressor screen

The genetic assay for the identification of new regulator of (p)ppGpp homeostasis in *E. coli* was carried out as follows. A pool of plasmids from the ASKA library (Kitagawa et al., 2005) was introduced by electroporation into *E. coli* MG1655 cells containing the pBbS2k-*relA*. The transformed cells were then spread on NA plates containing the appropriate antibiotics and the inducers aTc (0 or 100 ng/mL) and IPTG (0, 100, or 200 µM) for induction of pBbS2k-*relA* and pCA24N derivatives from the ASKA library, respectively. After 36 hr of incubation at 37°C, clones that were able to form colonies under these non-permissive conditions were chosen for the plasmid preparation and sequencing. The pool of plasmids from the ASKA library was obtained by Germain et al., 2019.

### Growth assay

The ability of *E. coli* cells to grow under specific conditions was tested as follows. Single colonies were inoculated into 2 mL of LB broth, supplemented with the appropriate antibiotic(s), and cultured at 37°C until stationary phase (~8 hr). The cultures were then serially diluted and spotted on NA, M9-glucose minimal medium, or SMG plates (Supplementary file 1) containing the appropriate antibiotic(s) and plasmid inducer(s). For growth assays on M9-glucose minimal medium or SMG, the cultures were washed twice and diluted in PBS before being serially diluted and spotted on agar plates. The cells were finally incubated at 37°C overnight or ~36 hr depending on whether they were spotted on NA or minimal medium, respectively.

### In vivo (p)ppGpp measurement

The levels of (p)ppGpp in the cells were determined as described by Germain et al., 2019. Briefly, 100 µL of ^32^P-labeled cell samples were taken at set times and added to 40 µL of ice-cold 21 M formic acid to stop the reactions. The mixtures were then placed on ice for 20 min before being centrifuged at 4°C for 20 min at 14,000 *g* to pellet cell debris and avoid them on chromatograms. 5 µL of each were loaded onto PEI-Cellulose thin layer chromatography (TLC) plates (Merck-Millipore) prior to ascending development with 1.5 M KH_2_PO_4_ solution (pH 3.4). Once fully developed, the TLC plates were dry at room temperature, separated from their upper part containing free ^32^P, and exposed overnight with a phosphor screen. The signals from the phosphor screen were then captured and quantified using an Amersham Typhoon Biomolecular Imager and ImageQuant TL 8.1 (GE Healthcare). We normalized the amount of (p)ppGpp to the total amount of G nucleotides observed in each sample, the total G being the sum of GTP, ppGpp, and pppGpp detected.

Regarding the preparation of the media, the labeling with ^32^P, and the induction of stress or starvation, they were carried out as follows. For overnight cultures, cells were grown in MOPS minimal medium (Neidhardt et al., 1974) supplemented with 0.2% glucose, 2 mM phosphate, and amino acids at 40 µg/mL (except for amino acid starvation experiment). Cultures were diluted 100 times in the same medium with 0.4 mM phosphate and incubated at 37°C with shaking. At an OD_600nm_ of ~0.5, they were diluted to an OD_600nm_ of 0.05, labeled with 150 µCi of ^32^P, and grew to an OD_600nm_ of ~0.20. At this point and 30 min later, inducers (100 ng/mL aTc or 1 mM IPTG) or 500 µg/mL L-valine were added for gene overexpression or induction of isoleucine starvation, respectively.

### Bacterial two-hybrid assay

In vivo protein-protein interactions were tested using the bacterial two-hybrid system (Karimova et al., 1998). Briefly, protein pairs were fused to the T18 and T25 domains of *B. pertussis* adenylate cyclase using the two compatible vectors pUT18C and pKT25, respectively. Co-transformed with the pairs of plasmids, single colonies of the *cya*-deficient strain BTH101 were inoculated into 2 mL of LB broth supplemented with ampicillin and kanamycin, and grown at 30°C to stationary phase (~12 hr). 5 µL of the cultures were then spotted on NA plates containing the two antibiotics and 40 µg/mL X-gal as a color reporter for β-galactosidase and adenylate cyclase activities. The cells were finally incubated at 30°C ~36 hr. To quantify the strength of the interaction, β-galactosidase activity was determined as described by Miller, 1992 using stationary phase culture (~12 hr).

### Screening for loss of interaction by *nirD* random mutagenesis

*nirD *DNA coding sequence was mutagenized randomly through low-fidelity GoTaq polymerase amplification from pUT18C-*nirD* plasmid using 441 and 443 oligonucleotides (Appendix 1—key resources table). After 30 PCR cycles, amplified DNA was purified, diluted to 1:100,000 and further amplified for 30 cycles. Purified DNA were cloned into pUT18C plasmid. BTH101 cells harboring pKT25*-relA* were then transformed with the resulting pUT18C-*nirD* mutated library and screened on X-Gal plates. White colonies were selected and sequenced.

### MG1655 *nirD^E50K^* construction

The chromosomal mutant strain *nirD^E50K^* was constructed by scarless mutagenesis using a two-step lambda Red system adapted from Blank et al., 2011. DNA fragments containing a chloramphenicol resistance marker and the meganuclease I-*SceI* recognition site were amplified by PCR using primers 629 and 630 with pWRG100 as template. The resulting PCR product was electroporated into MG1655 cells, which had induced lambda recombinase expression for 1 hr from plasmid pKD46 (Datsenko and Wanner, 2000). After 1 hr of phenotypic expression, the cells were plated on NA plates containing 25 µg/mL chloramphenicol. Selected colonies contained the *nirD::cat* I-*Sce*I allele, which was subsequently transduced in MG1655 cells harboring the pWRG99 plasmid (Blank et al., 2011). The mutant allele *nirD^E50K^* was amplified from pEG25-*nirD^E50K^* using primers 655 and 656. The PCR product was then electroporated into MG1655 *nirD::cat* I-*Sce*I expressing lambda recombinase from pWRG99 and after 1 hr of phenotypic expression, cells were serially diluted and plated on NA plates containing 100 µg/mL ampicillin and 1 µg/mL aTc. pWRG99 also encoded for the meganuclease I-*Sce*I under the control of an aTc-inducible promoter. The proper integration of the *nirD^E50K^* was confirmed by diagnostic PCR and sequencing.

### Growth curves

Growth of WT cells, Δ*nirD,* and *nirD^E50K^* mutants was measured under anaerobiosis as follows. Stationary-phase cultures were washed in PBS, diluted to an OD_600nm_ of ~0.03 in M9-glucose minimal medium without amino acids or supplement with 1 mM SMG in Hungate tubes, and air was evacuated and replaced with N_2_. The cultures were then incubated at 37°C and growth was followed by measurement of OD_600nm_. Although at least three independent experiments were performed, representative growth curves are shown. Indeed, day-to-day variations in medium composition shifted the curves slightly, but the sample-to-sample comparisons were invariable.

For the functional test of the NADH-dependent nitrite reductase activity, the cultures were prepared as described above except that they were diluted in a defined medium supplemented with 140 mM glycerol and 100 mM nitrate. The defined medium is composed of Na_2_HPO_4_ (60 mM), KH_2_PO_4_ (22 mM), NaCl (8 mM), MgSO_4_ (1 mM), CaCl_2_ (100 µM), thiamine (1 µg/mL), sodium molybdate (5 µM), sodium selenite (1 µM), and FeSO_4_ (10 µM).

### Protein expression and production

BL21 (DE3)-competent cells were transformed with the pEG25-*relA^N-terminal^*, pET28a(+)-*nirD* or pET28a(+)-*nirD^E50K^* and plated on selective NA. For RelA^N-terminal^ production, several colonies were picked up and inoculated into 100 mL LB containing 100 µg/mL ampicillin. The cultures were grown with shaking at 30°C overnight. Overnight cultures were dispersed as inoculum of 20 mL aliquots into 1 L Terrific Broth media with 100 µg/mL ampicillin and shaken at 30°C until OD_600nm_ ~0.5, then cells were induced with 0.5 mM IPTG and incubated at 30°C for 4 hr. For NirD and NirD^E50K^ production, several colonies were picked up and inoculated into 100 mL LB containing 50 µg/mL kanamycin. The cultures were grown with shaking at 30°C overnight. Overnight cultures were dispersed as inoculum of 20 mL aliquots into 1 L LB media with 50 µg/mL kanamycin and shaken at 30°C until OD_600nm _~0.5, then cells were induced with 0.5 mM IPTG and incubated at 30°C for 4 hr. Finally, cells were centrifuged at 9000 *g* for 20 min at 4°C. Dry cell pellet was stored at −80°C.

### Protein purification

For NirD and NirD^E50K^ purification, cells were suspended in lysis buffer (50 mM Tris-HCl [pH 8.0], 300 mM NaCl, 1 mM EDTA, 0.5 mg/mL lysozyme, 1 mM phenylmethylsulfonyl fluoride [PMSF], 20 µg/mL DNase, and 20 mM MgCl_2_), incubated for 1 hr at 4°C with gentle shaking, and then subjected to three cycles of French-press lysis steps. The soluble fraction was obtained by centrifugation for 20 min at 200,000 *g*. Recombinant proteins were purified by ion metal affinity chromatography using a 5 mL nickel (HiTrap^HP^) column on an ÄKTA pure 25 (GE Healthcare) pre-equilibrated in 50 mM Tris-HCl (pH 8.0), 300 mM NaCl, and 10 mM imidazole (buffer A). After several washes in buffer A, His-tagged proteins were eluted in buffer A supplemented with 250 mM imidazole and directly desalted using HiPrep 26/10 Desalting column pre-equilibrated with buffer A. The desalted proteins were mixed with 0.2 mg/mL of TEV protease, incubated for 2 hr at 25°C, and then loaded onto a HisTrap column pre-equilibrated in buffer A to retain TEV, TRX, uncleaved proteins, and contaminants. Untagged NirD or NirD^E50K^ were collected in the flow-through, concentrated on a Centricon (Millipore; cutoff of 3 kDa), and passed through a HiLoad 16/600 Superdex 200 column pre-equilibrated with 50 mM Tris-HCl (pH 8.0), 300 mM NaCl, 2 mM β-mercaptoethanol, and 2% glycerol. The purity of NirD preparations was assessed by SDS-PAGE (Figure 5—figure supplement 1B, C).

For RelA^N-terminal^ purification, cells were suspended in lysis buffer (50 mM Tris-HCl [pH 8.0], 500 mM NaCl, 10 mM imidazole, 2 mM β-mercaptoethanol, 0.5% CHAPS, 2% glycerol, 1 mM EDTA, 0.5 mg/mL lysozyme, 1 mM PMSF, 20 µg/mL DNase, and 20 mM MgCl_2_), incubated for 1 hr at 4°C with gentle shaking, and then subjected to three cycles of French-press lysis steps. The soluble fraction was obtained by centrifugation for 20 min at 200,000 *g*. Recombinant proteins were purified by ion metal affinity chromatography using a 5 mL nickel (HiTrap^HP^) column on an ÄKTA pure 25 (GE Healthcare) pre-equilibrated with equilibrium buffer (50 mM Tris-HCl [pH 8.0], 500 mM NaCl, 10 mM imidazole, 2 mM β-mercaptoethanol, and 2% glycerol), eluted in elution buffer (50 mM Tris-HCl [pH 8.0], 250 mM NaCl, 500 mM imidazole, 2 mM β-mercaptoethanol, and 2% glycerol), and directly desalted using HiPrep 26/10 Desalting column pre-equilibrated with buffer containing 50 mM Tris-HCl (pH 8.0), 500 mM NaCl, 500 mM KCl, 2 mM β-mercaptoethanol, and 2% glycerol. Desalted proteins were concentrated on a Centricon (Millipore; cutoff of 10 kDa) and then subjected to SEC purification using a HiLoad 16/600 Superdex 200 column pre-equilibrated with 50 mM Tris-HCl (pH 8.0), 500 mM NaCl, 500 mM KCl, 2 mM β-mercaptoethanol, and 2% glycerol. The purity of RelA preparations was assessed by SDS-PAGE (Figure 5—figure supplement 1A).

### Protein-protein interaction analysis by SEC

Purified RelA^N-terminal^ and NirD were either alone or pre-mixed in a 1:1 ratio (10 µM each) in buffer containing 50 mM Tris-HCl (pH 8.0), 500 mM NaCl, 500 mM KCl, 2 mM β-mercaptoethanol, and 2% glycerol prior separation on a Superdex 200 10/300 GL using an ÄKTA pure 25 apparatus (GE Healthcare) pre-equilibrated with the same buffer. Elution fractions were then analyzed with SDS-PAGE.

### Biolayer interferometry

NirD or NirD^E50K^ were biotinylated using the EZ-Link NHS-PEG4-Biotin (Thermo Scientific) at 4°C for 2 hr. The reaction was stopped by removing the excess of the biotin using a Zeba Spin Desalting column (Thermo Scientific). BLI studies were performed at 25°C using the Blitz apparatus (FortéBio) with shaking at 2200 rpm with the following steps: 30 s baseline, 120 s association, and 120 s dissociation. Streptavidin biosensor tips (FortéBio) were hydrated with 0.350 mL of 50 mM Tris-HCl (pH 8.0), 500 mM NaCl, 500 mM KCl, 2 mM β-mercaptoethanol, and 2% glycerol for 10 min and then loaded with 3 µM of biotinylated NirD or NirD^E50K^ in the same buffer. The biosensors were then incubated with 10 µg/mL biocytin in interaction buffer (50 mM Tris-HCl [pH 8.0], 500 mM NaCl, 500 mM KCl, 2 mM β-mercaptoethanol, 2% glycerol, and 1 mg/mL) for 90 s to avoid the non-specific binding RelA^N-terminal^ to the streptavidin biosensors. To study the binding of NirD or NirD^E50K^ to RelA^N-terminal^, increasing concentrations RelA^N-terminal ^(1–30 µM) in interaction buffer were used and the association and dissociation phases were monitored, respectively. In all experiments, the BLItz Pro Software performed a reference subtraction of the RelA^N-terminal^ protein response on the uncoated biosensors for each tested concentration. The dissociation constant (K_D_), that is affinity of NirD to RelA^N-terminal^, was estimated using the GraphPad Prism 5.0 software on the basis of the steady-state-level responses in nm at equilibrium. The K_D_ was estimated by plotting on x axis the different concentrations and the different responses of RelA^N-terminal^ at the saturation (10 s before the end of the association) on the y axis. For K_D_ calculation, a nonlinear regression fit for xy analysis was used and one site (specific binding) as a model [corresponding to the equation y=Bmax*x/(K_D_+x)].

### Isothermal titration calorimetry

ITC was performed to determine the affinity between NirD and RelA^N-terminal^. In a typical setup, RelA^N-terminal^ (30 µM) in buffer containing 50 mM Tris-HCl (pH 8.0), 500 mM NaCl, 500 mM KCl, 2 mM β-mercaptoethanol, and 2% glycerol was placed in the cell, and NirD (or NirD^E50K^) ligand (300 μM in the same buffer) was placed in the titration syringe. All experiments were carried out at 25°C with a stirring speed of 750 rpm and a reference power of 5 µcal/s using the MicroCal PEAQ-ITC (Malvern Panalytical) with 19 injections (0.4 µL for the initial injection and 2 µL for the next 18). Each injection lasted for 4 s with a space of 150 s between each injection. ITC measures the energy released or absorbed by a chemical reaction, and the energy of the interaction is proportional to the amount of ligand that binds to the protein. These heat pulses are recorded for each injection and then integrated with respect to time and are normalized to the concentration of the reaction to generate kcal/mol versus the molar ratio (ligand/sample). The enthalpy of the reaction is obtained, and with a ‘One Set of Sites’ model in the PEAQ-ITC Analysis Software, the K_D_ and stoichiometry is determined. The fitted offset option in the PEAQ-ITC Analysis Software was used to correct for the heat of the dilution.

### pppGpp synthesis assay

The pppGpp synthetic activity of RelA^N-terminal^ was assayed at 37°C in a buffer containing 50 mM Tris-HCl (pH 8.0), 300 mM NaCl, 2 mM β-mercaptoethanol, 2% glycerol, and 15 mM Mg^2+^. Typically, reaction mixtures contained 8 mM ATP, 6 mM GTP and [α-^32^P]GTP, 1 µM RelA^N-terminal^ with or without NirD (or NirD^E50K^) at concentrations ranging from 0.5 to 16 µM, and reactions were started by adding pre-warmed nucleotides to the protein(s). During the time course of the reaction, 20 µL samples were taken and added to 8 µL of ice-cold 21 M formic acid to stop the reactions. The time points were resolved by TLC, and radio-labeled nucleotides were quantified as described in the ‘In vivo (p)ppGpp measurement’ section.

## Data Availability

All data generated or analysed during this study are included in the manuscript and supporting files. Source data files have been provided for Figure 1, Figure 2, Figure 3, Figure 4, Figure 5, Figure 6, Figure 3-figure supplement 1, Figure 4-figure supplement 1 and Figure 5-figure supplement 1.

## Additional files

Supplementary file 1. Media used in this work.

Transparent reporting form

## Appendix 1

**Appendix 1—key resources table:**

Reagent type (species) or resource	Designation	Source or reference	Identifiers	Additional information
strain, strain background (*Escherichia coli*)	BTH101	Karimova et al., 1998 doi: 10.1073/pnas.95.10.5752		F^–^ *cya-99 araD139 galE15 galK16 rpsL*(*Str*^*R*^) *hsdR2 mcrA1 mcrB1 relA1*
strain, strain background (*E. coli*)	BL21 (DE3)	New England Biolabs		B F^–^ *ompT gal*
strain, strain background (*E. coli*)	MG1655; WT	Lab collection		Wild-type
strain, strain background (*E. coli*)	*nirD*^*E50K*^	This paper		MG1655 *nirD*^*E50K*^
strain, strain background (*E. coli*)	Δ*nirD*	This paper		MG1655 *nirD::FRT*; P1 from KEIO collection (resistance cassette has been flipped out) (Baba et al., 2006)
strain, strain background (*E. coli*)	Δ*relA*	Germain et al., 2019 doi: 10.1038/s41467-019-13764-4		MG1655 *relA::FRT*
strain, strain background (*E. coli*)	Δ*relA spoT*	This paper		MG1655 relA::FRT spoT207::cat; P1 transduction from CF1693 (Baba et al., 2006) in MG1655 *relA::FRT*
recombinant DNA reagent	pBbS2k-*rfp*	Lee et al., 2011 doi: 10.1186/1754-1611-5-12		Kan^R^, P_tet_, *rfp*
recombinant DNA reagent	pBbS2k-*relA*	This paper		Kan^R^, P_tet_, *relA*; primers 16/17; Maisonneuve lab
recombinant DNA reagent	pBbS2k-*relA* (sd4)	This paper		Kan^R^, P_tet_, sd4, *relA*; primers 163/17; Maisonneuve lab
recombinant DNA reagent	pBbS2k-*relA*^*N-terminal*^	This paper		Kan^R^, P_tet_, *relA*^*N-terminal*^; primers 16/557; Maisonneuve lab
recombinant DNA reagent	pEG25	Germain et al., 2019 doi: 10.1038/s41467-019-13764-4		Amp^R^, P_T5-lac_
recombinant DNA reagent	pEG25-*nirD*	This paper		Amp^R^, P_T5-lac_, *nirD*; primers 386/387; Maisonneuve lab
recombinant DNA reagent	pEG25-*spoT* (sd8U)	Germain et al., 2019 doi: 10.1038/s41467-019-13764-4		Amp^R^, P_T5-lac_, sd8U, *spoT*
recombinant DNA reagent	pEG25-*nirD*^*E50K*^	This paper		Amp^R^, P_T5-lac_, *nirD*^*E50K*^; primers 386/387; Maisonneuve lab
recombinant DNA reagent	pEG25-*nirD* (WTsd)	This paper		Amp^R^, P_T5-lac_, WTsd, *nirD*; primers 555/387; Maisonneuve lab
recombinant DNA reagent	pEG25-*nirBD* (WTsd)	This paper		Amp^R^, P_T5-lac_, WTsd, *nirBD*; primers 553/544; Maisonneuve lab
recombinant DNA reagent	pEG25-*relA*^*N-terminal*^	Germain et al., 2019 doi: 10.1038/s41467-019-13764-4		Amp^R^, P_T5-lac_, *relA*^*N-terminal*^
recombinant DNA reagent	pKD46	Datsenko and Wanner, 2000 doi: 10.1073/pnas.120163297		Amp^R^, P_ara_, λ Red
recombinant DNA reagent	pKT25	Karimova et al., 1998 doi: 10.1073/pnas.95.10.5752		Kan^R^
recombinant DNA reagent	pKT25-*zip*	Karimova et al., 1998 doi: 10.1073/pnas.95.10.5752		Kan^R^, *zip*
recombinant DNA reagent	pKT25-*relA*	Germain et al., 2019 doi: 10.1038/s41467-019-13764-4		Kan^R^, *relA*
recombinant DNA reagent	pKT25-*spoT*	Germain et al., 2019 doi: 10.1038/s41467-019-13764-4		Kan^R^, *spoT*
recombinant DNA reagent	pKT25-*nirB*	This paper		Kan^R^, *nirB*; primers 617/618; Maisonneuve lab
recombinant DNA reagent	pKT25-*relA*^*1-663*^	This paper		Kan^R^, *relA*^*1-663*^; primers 497/502; Maisonneuve lab
recombinant DNA reagent	pKT25-*relA*^*1-580*^	This paper		Kan^R^, *relA*^*1-580*^; primers 497/501; Maisonneuve lab
recombinant DNA reagent	pKT25-*relA*^*1-470*^	This paper		Kan^R^, *relA*^*1-470*^; primers 497/500; Maisonneuve lab
recombinant DNA reagent	pKT25-*relA*^*N-terminal*^	This paper		Kan^R^, *relA*^*N-terminal*^; primers 497/499; Maisonneuve lab
recombinant DNA reagent	pKT25-*relA*^*1-181*^	This paper		Kan^R^, *relA*^*1-181*^; primers 497/498; Maisonneuve lab
recombinant DNA reagent	pKT25-*relA*^*181-744*^	This paper		Kan^R^, *relA*^*181-744*^; primers 531/533; Maisonneuve lab
recombinant DNA reagent	pKT25-*spoT*^*N-terminal*^	Germain et al., 2019 doi: 10.1038/s41467-019-13764-4		Kan^R^, *relA*^*N-terminal*^
recombinant DNA reagent	pUT18C	Karimova et al., 1998 doi: 10.1073/pnas.95.10.5752		Amp^R^
recombinant DNA reagent	pUT18C-*zip*	Karimova et al., 1998 doi: 10.1073/pnas.95.10.5752		Amp^R^, *zip*
recombinant DNA reagent	pUT18C-*nirD*	This paper		Amp^R^, *nirD*; primers 441/443; Maisonneuve lab
recombinant DNA reagent	pUT18C-*nirD*^*E50K*^	This paper		Amp^R^, *nirD*^*E50K*^; primers 441/443; Maisonneuve lab
recombinant DNA reagent	pET28a(+)	Germain et al., 2019 doi: 10.1038/s41467-019-13764-4		Kan^R^, Trx, His
recombinant DNA reagent	pET28a(+)-*nirD*	This paper		Kan^R^, Trx, His, TEV, *nirD*; primers 558/544; Maisonneuve lab
recombinant DNA reagent	pET28a(+)-*nirD*^*E50K*^	This paper		Kan^R^, Trx, His, TEV, *nirD*^*E50K*^; primers 558/544; Maisonneuve lab
recombinant DNA reagent	pWRG99	Blank et al., 2011 doi: 10.1371/journal.pone.0015763		pKD46 with Ptet, I-SceI
recombinant DNA reagent	pWRG100	Karimova et al., 1998 doi: 10.1073/pnas.95.10.5752 doi: 10.1371/journal.pone.0015763		pKD3 with I-*Sce*I recognition site
sequence-based reagent	Primer 16	This paper		CCCCGAATTCGTCGACTCAAGGAGGTTTTATAAATGGTTGCGGTAAGAAGTGCA; restriction enzyme EcoRI; Maisonneuve lab
sequence-based reagent	Primer 17	This paper		CCCCGGATCCCTAACTCCCGTGCAACCGAC; restriction enzyme BamHI; Maisonneuve lab
sequence-based reagent	Primer 163	This paper		CCCCGAATTCGTCGACTCAAGGATTAAATGGTTGCGGTAAGAAGTGCA; restriction enzyme EcoRI; Maisonneuve lab
sequence-based reagent	Primer 386	This paper		CCCCGAATTCGTCGACTCAAGGAGGTTTTATAAATGAGCCAGTGGAAAGACAT; restriction enzyme EcoRI; Maisonneuve lab
sequence-based reagent	Primer 387	This paper		CCCCGGATCCTTAACCGCGCAGCTGCACCA; restriction enzyme BamHI; Maisonneuve lab
sequence-based reagent	Primer 441	This paper		CCCCTCTAGAAATGAGCCAGTGGAAAGACAT; restriction enzyme XbaI; Maisonneuve lab
sequence-based reagent	Primer 443	This paper		CCCCGGTACCTTAACCGCGCAGCTGCACCA; restriction enzyme KpnI; Maisonneuve lab
sequence-based reagent	Primer 497	This paper		CCCCTCTAGAAATGGTTGCGGTAAGAAGTGC; restriction enzyme XbaI; Maisonneuve lab
sequence-based reagent	Primer 498	This paper		CCCCGGTACCGATGTTGGTACACTCTTTTG; restriction enzyme KpnI; Maisonneuve lab
sequence-based reagent	Primer 499	This paper		CCCCGGTACCTTCGTCGAGCATTTCGCCGG; restriction enzyme KpnI; Maisonneuve lab
sequence-based reagent	Primer 500	This paper		CCCCGGTACCGTTCGGCTGTTTCTGGGTGA; restriction enzyme KpnI; Maisonneuve lab
sequence-based reagent	Primer 501	This paper		CCCCGGTACCAAGTTGCTTCAGCGCGGCGG; restriction enzyme KpnI; Maisonneuve lab
sequence-based reagent	Primer 502	This paper		CCCCGGTACCGGCGGAGTAGCTCTCACCCC; restriction enzyme KpnI; Maisonneuve lab
sequence-based reagent	Primer 531	This paper		CCCCTCTAGAATACGCACCGCTGGCTAACCG; restriction enzyme XbaI; Maisonneuve lab
sequence-based reagent	Primer 533	This paper		CCCCGGTACCCTAACTCCCGTGCAACCGAC; restriction enzyme KpnI; Maisonneuve lab
sequence-based reagent	Primer 544	This paper		CCCCAAGCTTTTAACCGCGCAGCTGCACCA; restriction enzyme HindIII; Maisonneuve lab
sequence-based reagent	Primer 553	This paper		CCCCGAATTCAATAGAAAAGAAATCGAGGCAAAAATGAGCAAAGTCAGACTCGC; restriction enzyme EcoRI; Maisonneuve lab
sequence-based reagent	Primer 555	This paper		CCCCGAATTCAGTAACTCTGGTGGAGGACAACGCATGAGCCAGTGGAAAGACAT; restriction enzyme EcoRI; Maisonneuve lab
sequence-based reagent	Primer 557	This paper		CCCCGGATCCTTATTCGTCGAGCATTTCGCCGG; restriction enzyme BamHI; Maisonneuve lab
sequence-based reagent	Primer 558	This paper		CCCCGGATCCGAGAACCTGTACTTCCAATCAATGAGCCAGTGGAAAGACAT; restriction enzyme BamHI; Maisonneuve lab
sequence-based reagent	Primer 617	This paper		CCCCTCTAGAAATGAGCAAAGTCAGACTCGC; restriction enzyme XbaI; Maisonneuve lab
sequence-based reagent	Primer 618	This paper		CCCCGGTACCTCATGCGTTGTCCTCCACCA; restriction enzyme KpnI; Maisonneuve lab
sequence-based reagent	Primer 629	This paper		ATGAGCCAGTGGAAAGACATCTGCAAAATCGATGACATCCTGCCTGAAACCGCCTTACGCCCCGCCCTGC; Maisonneuve lab
sequence-based reagent	Primer 630	This paper		TTAACCGCGCAGCTGCACCACGCCGTCTTTCACTCGCGCTTCGTAATGTTCTAGACTATATTACCCTGTT; Maisonneuve lab
sequence-based reagent	Primer 655	This paper		ATGAGCCAGTGGAAAGACAT; Maisonneuve lab
sequence-based reagent	Primer 656	This paper		TTAACCGCGCAGCTGCACCA; Maisonneuve lab
chemical compound, drug	[α^32^P]GTP	PerkinElmer	Catalog number: BLU006H250UC	
chemical compound, drug	^32^P	PerkinElmer	Catalog number: NEX053H001MC	
chemical compound, drug	EZ-Link NHS-PEG4-Biotin	Thermo Scientific	Catalog number: 21330	
software, algorithm	BLItz Pro Software		https://www.sartorius.com/en/products/protein-analysis	
software, algorithm	MicroCal PEAQ-ITC Analysis Software	Malvern Panalytical	https://www.malvernpanalytical.com/fr/products/product-range/microcal-range/microcal-itc-range/microcal-peaq-itc	
software, algorithm	Prism 5.0 software	GraphPad	https://www.graphpad.com/scientific-software/prism/	
other	HiLoad 16/600 Superdex 200 column	GE Healthcare	Catalog number: GE28-9893-35	
other	HiPrep 26/10 Desalting column	GE Healthcare	Catalog number: GE17-5087-01	
other	HisTrap column	GE Healthcare	Catalog number: GE17-5248-02	
other	Streptavidin Biosensors	FortéBio	Catalog number: 18-5117	
other	Superdex 200 10/300 GL	GE Healthcare	Catalog number: GE28-9909-44	
other	Zeba Spin Desalting column	Thermo Scientific	Catalog number: 89882	
