# Supplementary figures and images for: NirD curtails the stringent response by inhibiting RelA activity in Escherichia coli
Source: eLife. 2021 Jul 29;10:e64092. doi: 10.7554/eLife.64092 (PMC8321558; doi:10.7554/eLife.64092)

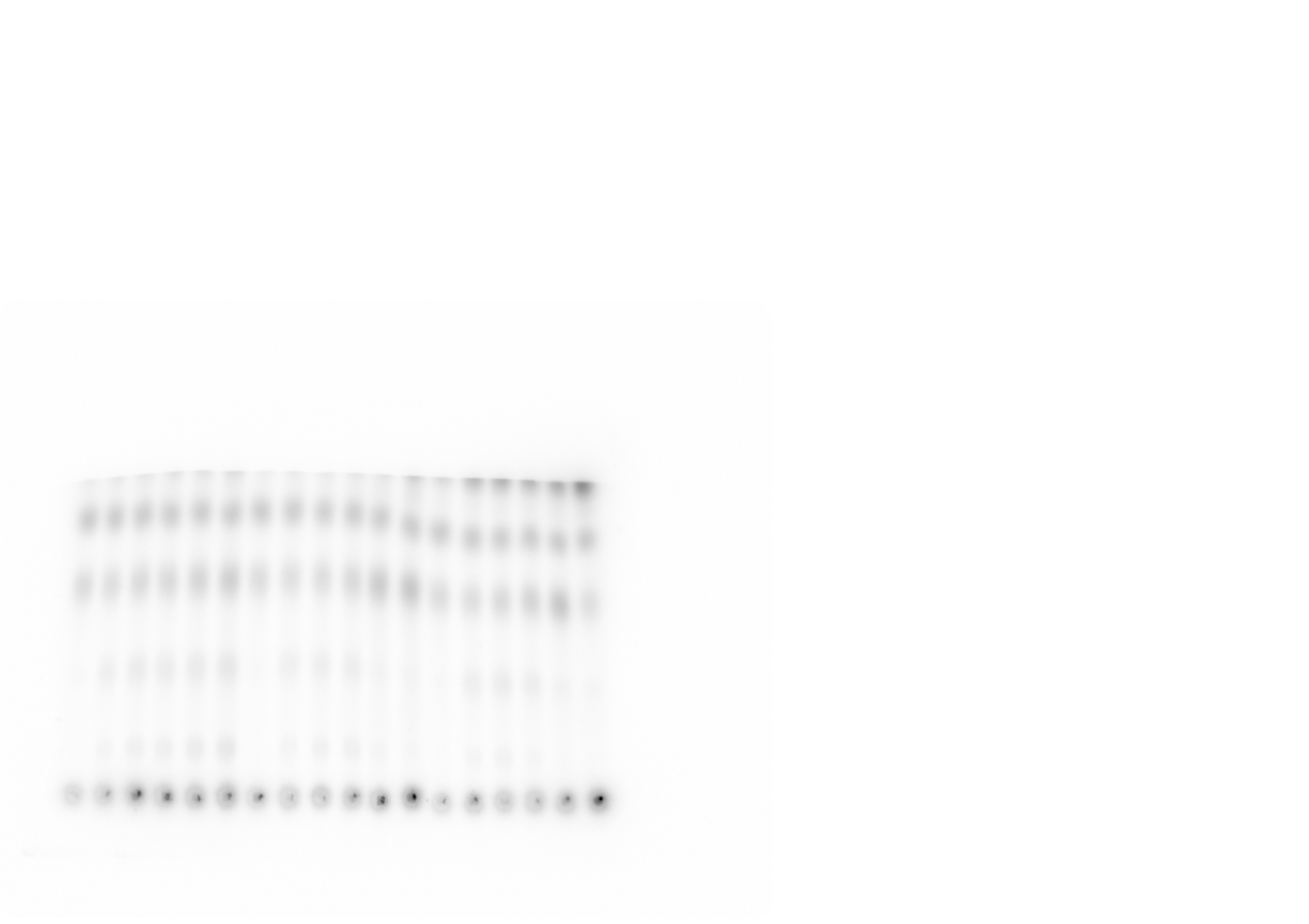

Supplement: Figure 1—source data 1. [file elife-64092-fig1-data1.zip › Figure 1-source data 1 (raw autoradiogram).gel]

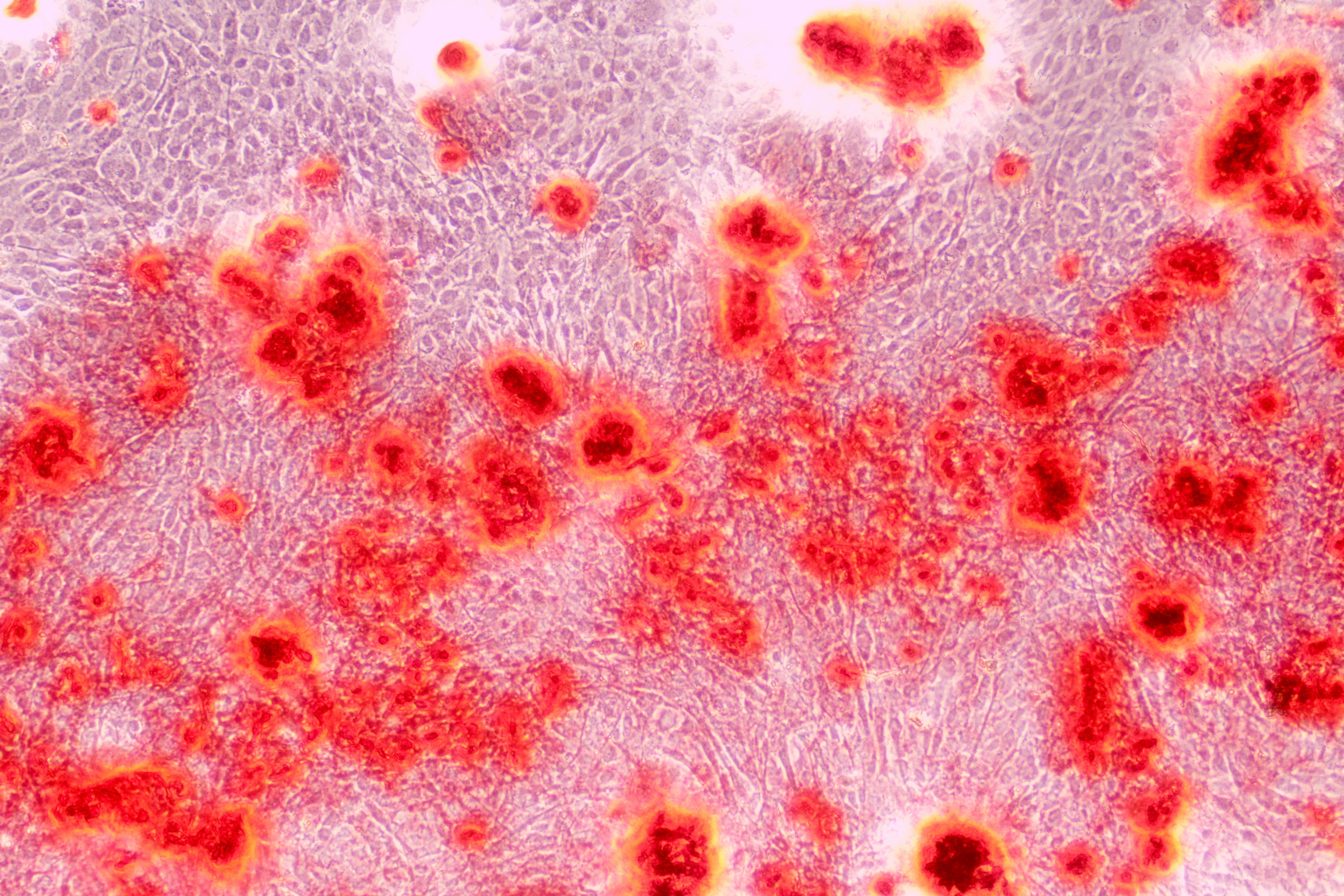

Supplement: Figure 1—source data 2. — The amount of (p)ppGpp was normalized to total amount of G nucleotides observed in each sample. Total G is the sum of GTP and (p)ppGpp detected. The source data are provided for the relative levels of (p)ppGpp in Figure 1D, which are represented as the means and SDs of three independent experiments. [file elife-64092-fig1-data2.zip › Figure 1-source data 2/100×/0.5μM.png]

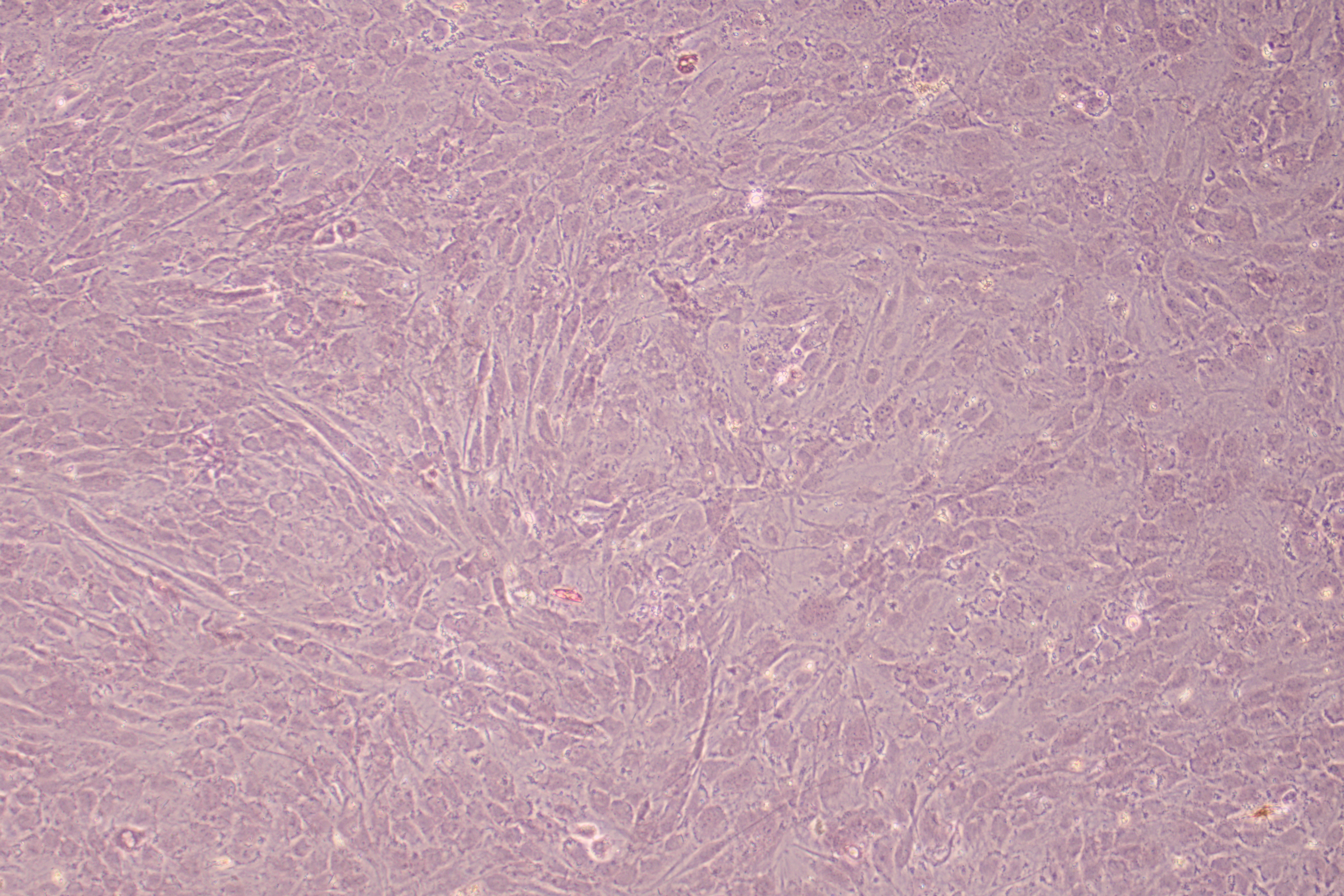

Supplement: Figure 1—source data 2. — The amount of (p)ppGpp was normalized to total amount of G nucleotides observed in each sample. Total G is the sum of GTP and (p)ppGpp detected. The source data are provided for the relative levels of (p)ppGpp in Figure 1D, which are represented as the means and SDs of three independent experiments. [file elife-64092-fig1-data2.zip › Figure 1-source data 2/100×/0.png]

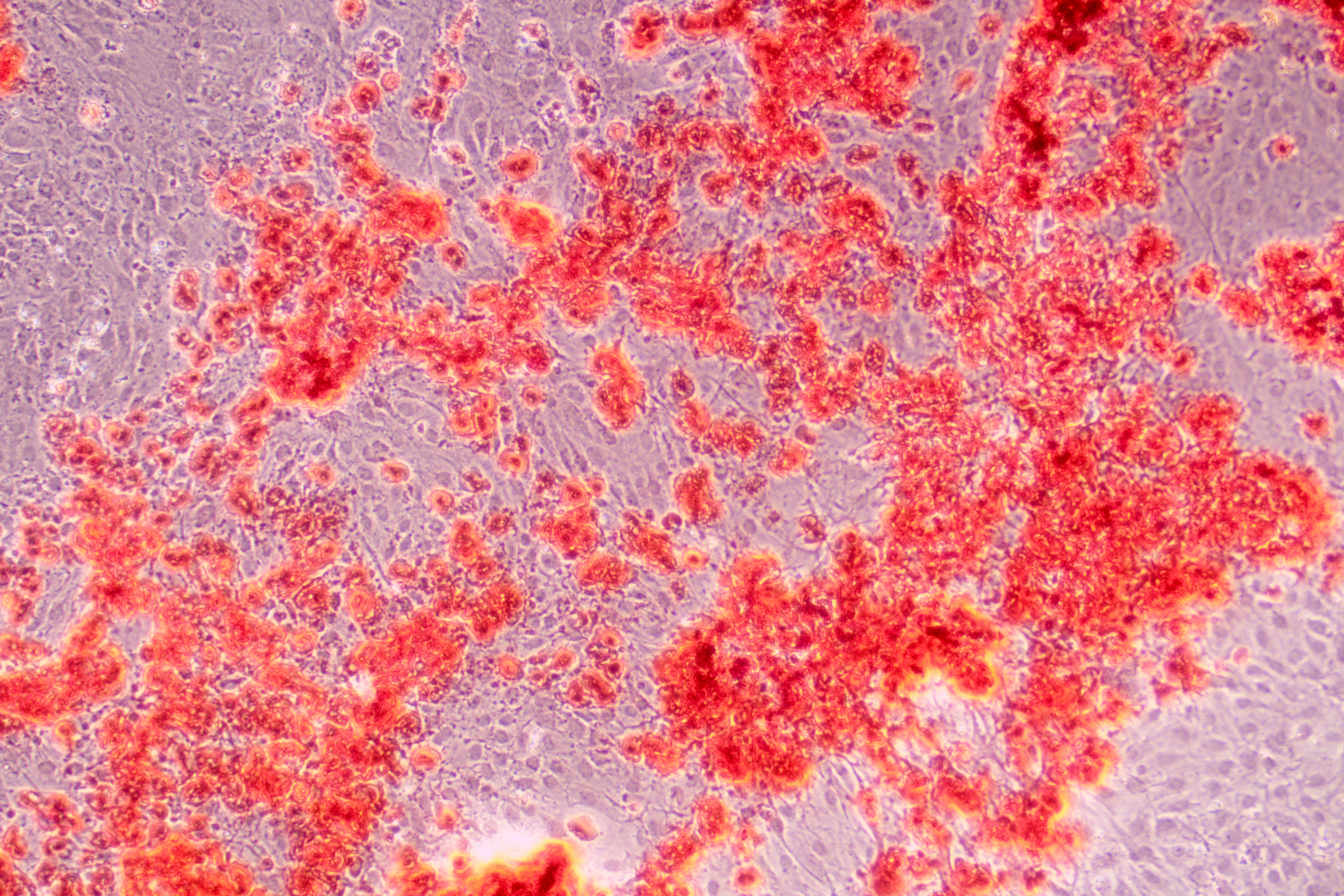

Supplement: Figure 1—source data 2. — The amount of (p)ppGpp was normalized to total amount of G nucleotides observed in each sample. Total G is the sum of GTP and (p)ppGpp detected. The source data are provided for the relative levels of (p)ppGpp in Figure 1D, which are represented as the means and SDs of three independent experiments. [file elife-64092-fig1-data2.zip › Figure 1-source data 2/100×/1μM.png]

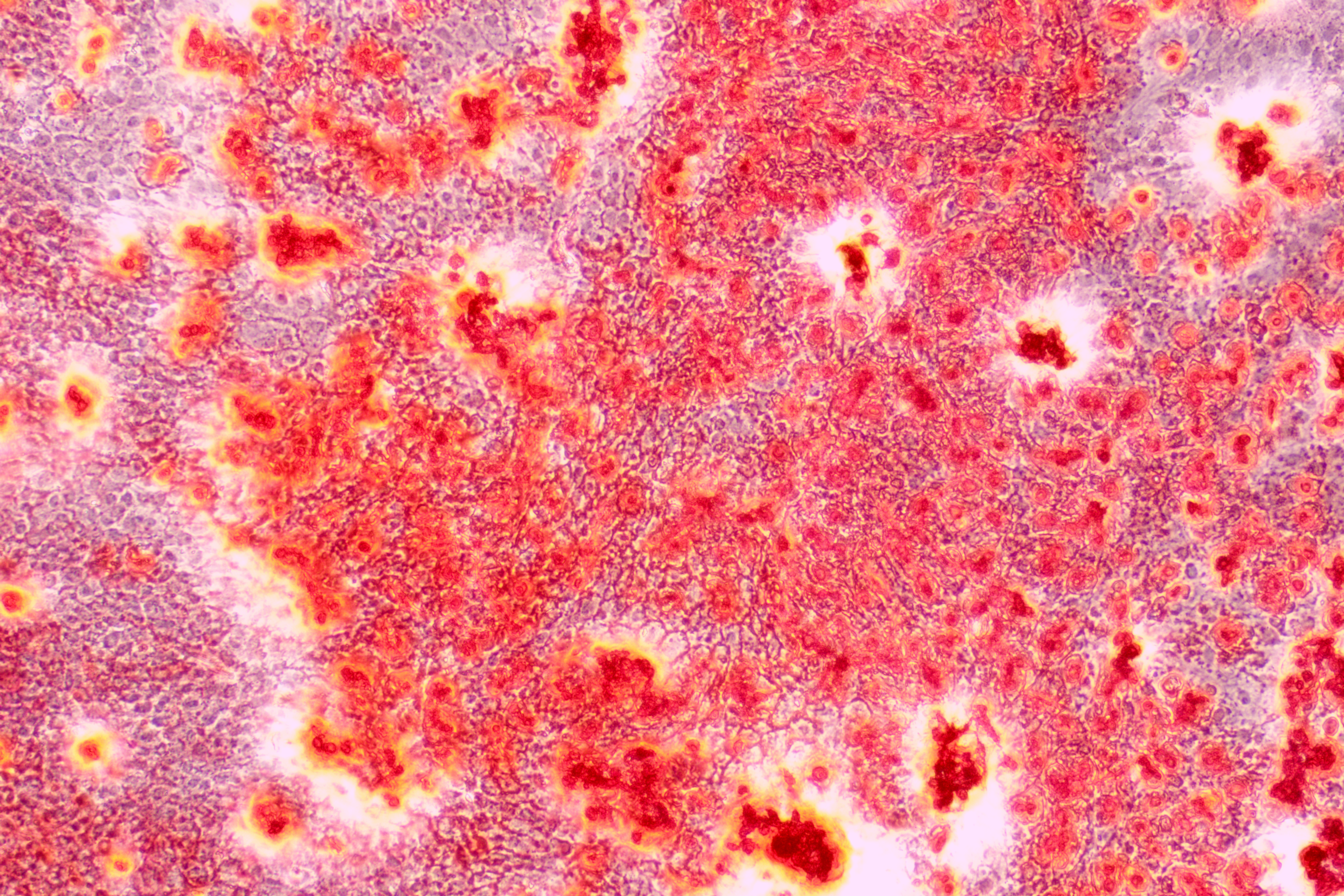

Supplement: Figure 1—source data 2. — The amount of (p)ppGpp was normalized to total amount of G nucleotides observed in each sample. Total G is the sum of GTP and (p)ppGpp detected. The source data are provided for the relative levels of (p)ppGpp in Figure 1D, which are represented as the means and SDs of three independent experiments. [file elife-64092-fig1-data2.zip › Figure 1-source data 2/100×/5μM.png]

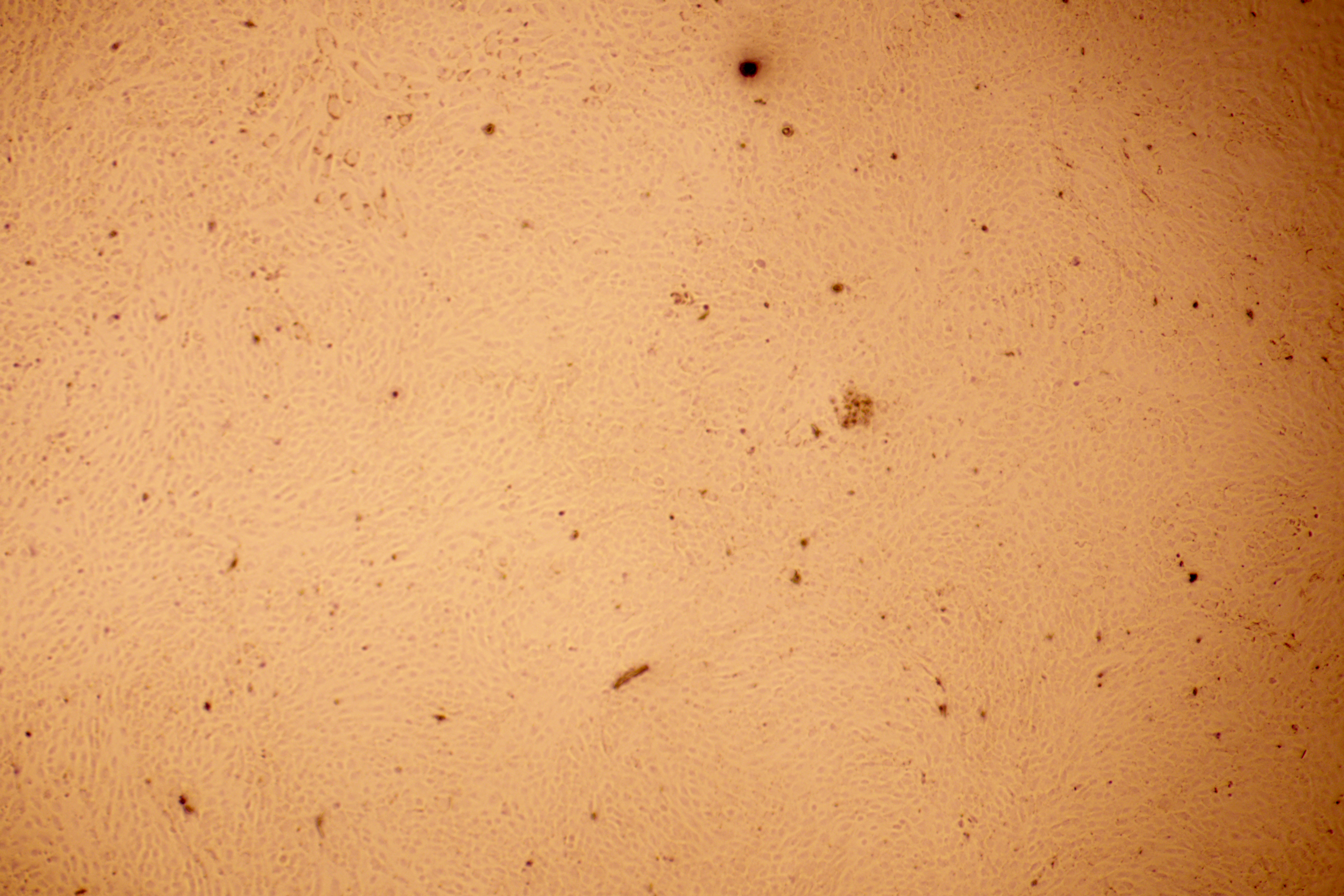

Supplement: Figure 1—source data 2. — The amount of (p)ppGpp was normalized to total amount of G nucleotides observed in each sample. Total G is the sum of GTP and (p)ppGpp detected. The source data are provided for the relative levels of (p)ppGpp in Figure 1D, which are represented as the means and SDs of three independent experiments. [file elife-64092-fig1-data2.zip › Figure 1-source data 2/40×/0.5 μM.png]

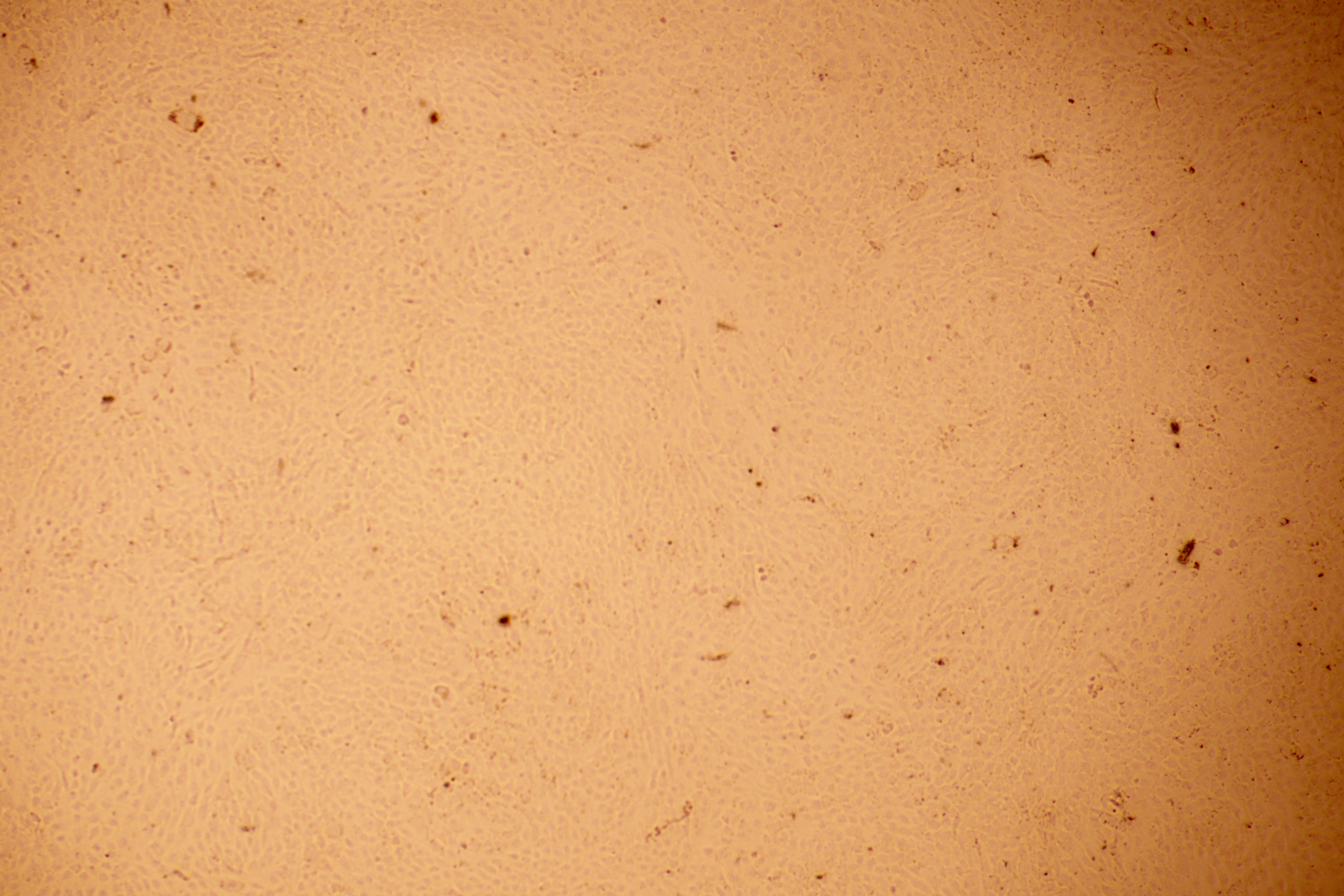

Supplement: Figure 1—source data 2. — The amount of (p)ppGpp was normalized to total amount of G nucleotides observed in each sample. Total G is the sum of GTP and (p)ppGpp detected. The source data are provided for the relative levels of (p)ppGpp in Figure 1D, which are represented as the means and SDs of three independent experiments. [file elife-64092-fig1-data2.zip › Figure 1-source data 2/40×/0.png]

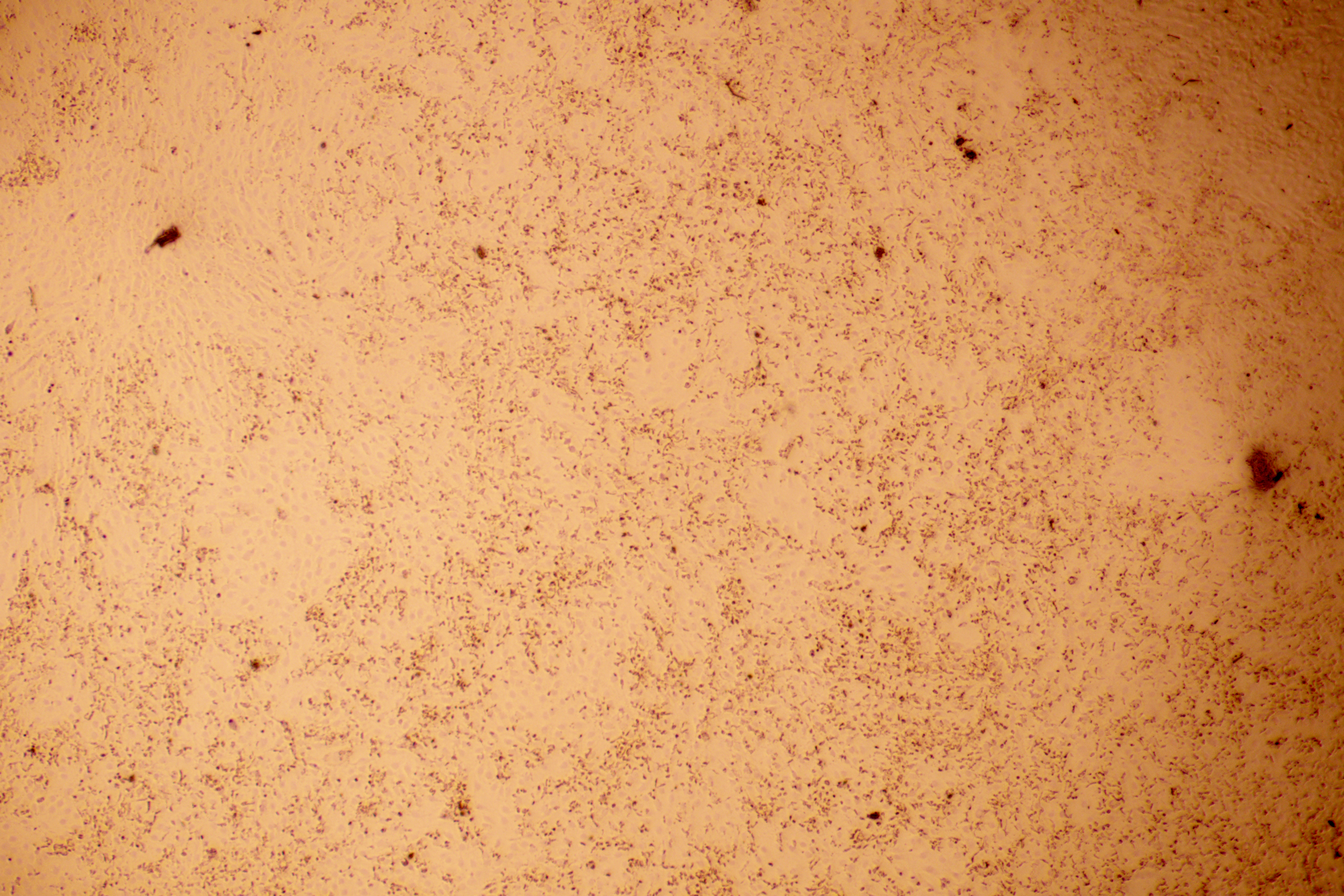

Supplement: Figure 1—source data 2. — The amount of (p)ppGpp was normalized to total amount of G nucleotides observed in each sample. Total G is the sum of GTP and (p)ppGpp detected. The source data are provided for the relative levels of (p)ppGpp in Figure 1D, which are represented as the means and SDs of three independent experiments. [file elife-64092-fig1-data2.zip › Figure 1-source data 2/40×/1 μM.png]

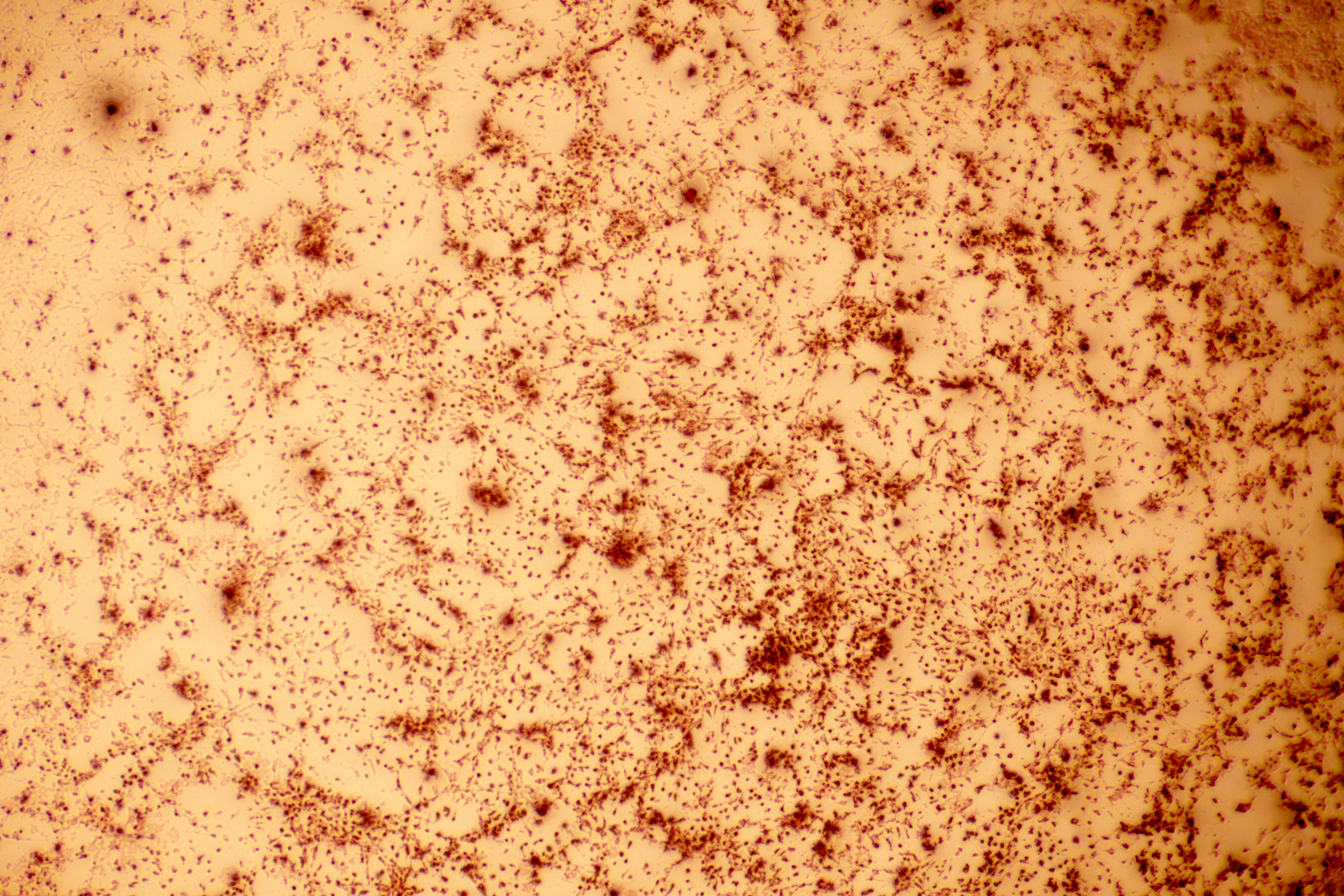

Supplement: Figure 1—source data 2. — The amount of (p)ppGpp was normalized to total amount of G nucleotides observed in each sample. Total G is the sum of GTP and (p)ppGpp detected. The source data are provided for the relative levels of (p)ppGpp in Figure 1D, which are represented as the means and SDs of three independent experiments. [file elife-64092-fig1-data2.zip › Figure 1-source data 2/40×/5 μM.png]

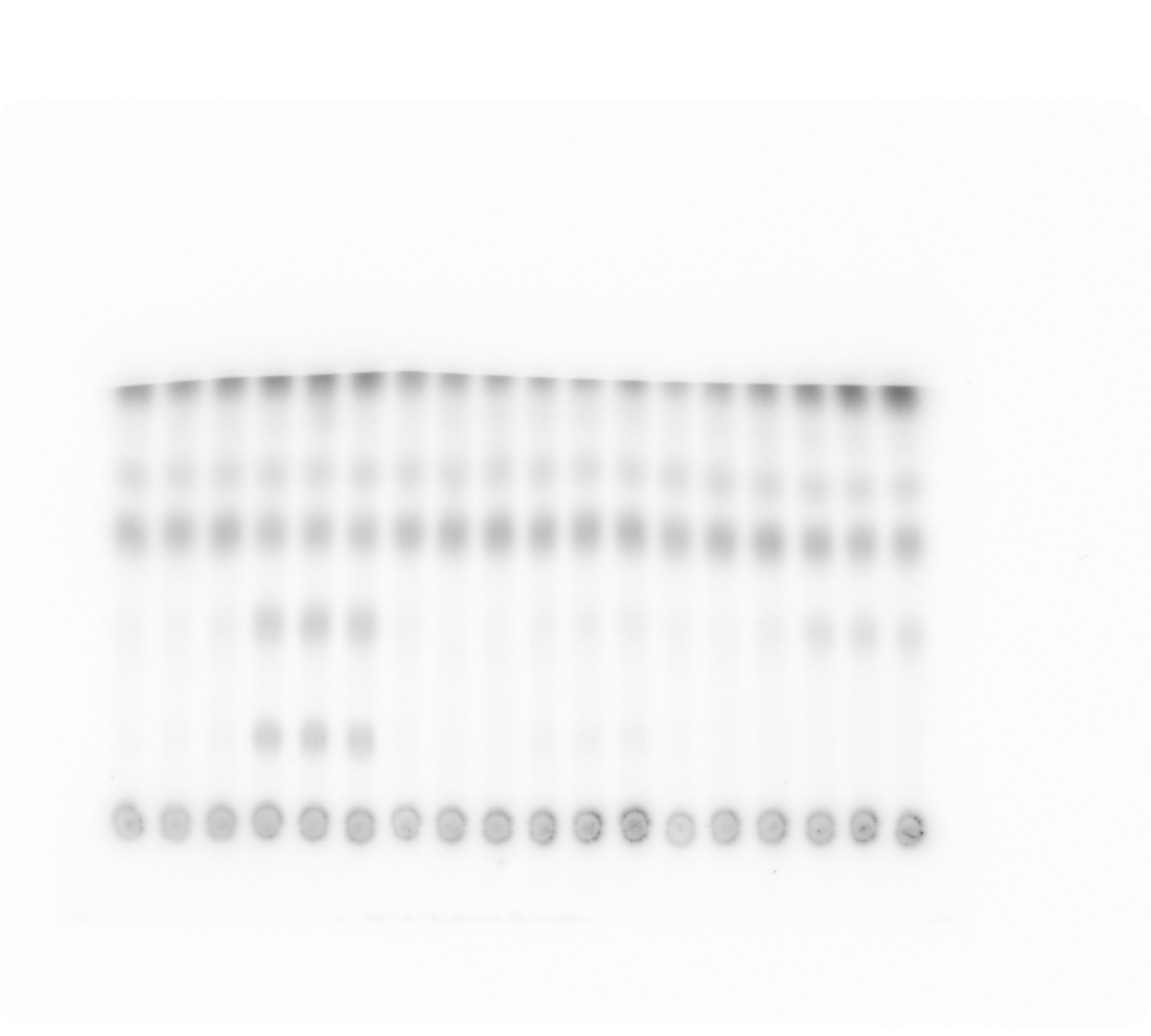

Supplement: Figure 2—source data 1. [file elife-64092-fig2-data1.zip › Figure 2-source data 1 (raw autoradiogram).gel]

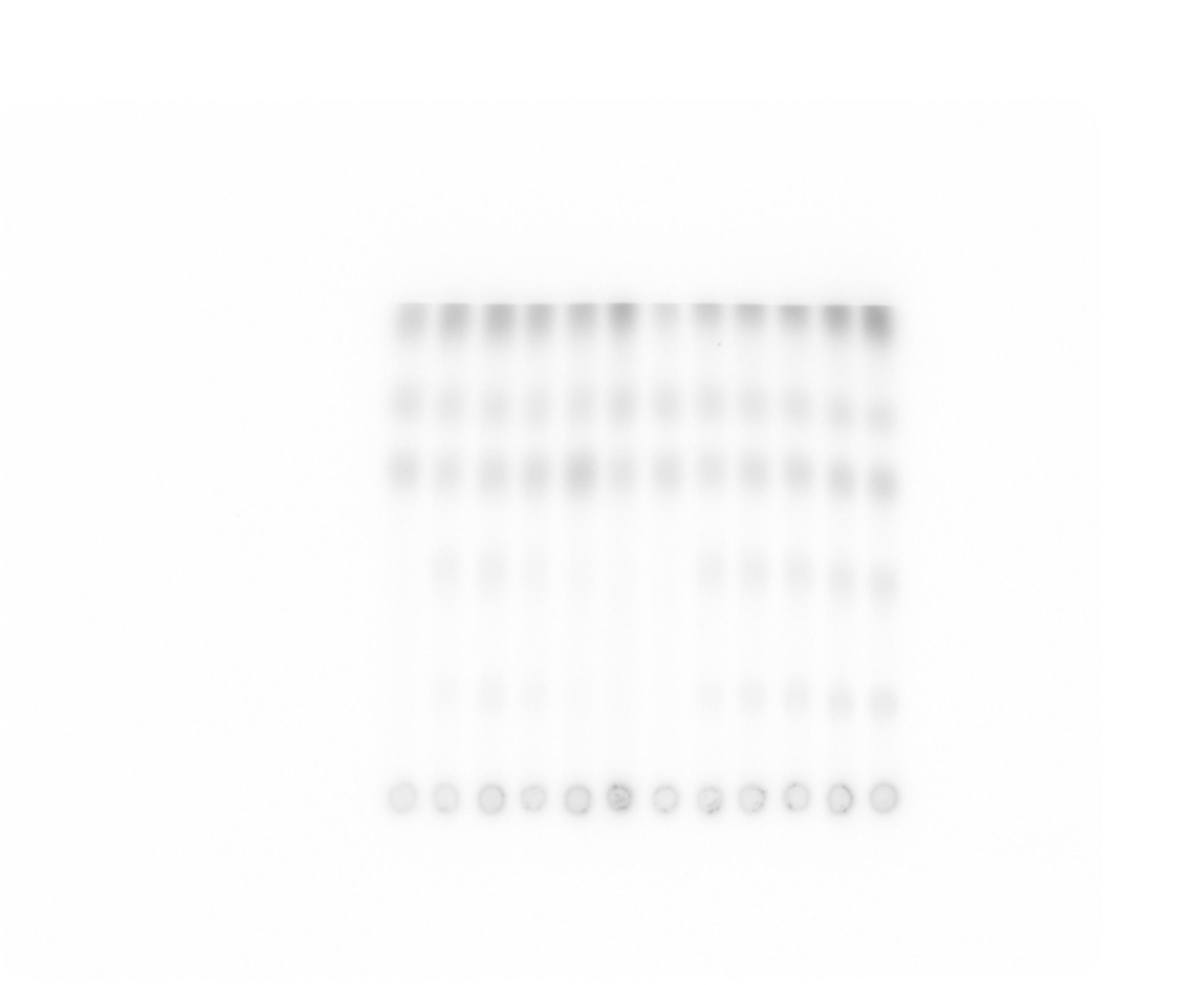

Supplement: Figure 3—source data 2. [file elife-64092-fig3-data2.zip › Figure 3-source data 2 (raw autoradiogram).gel]

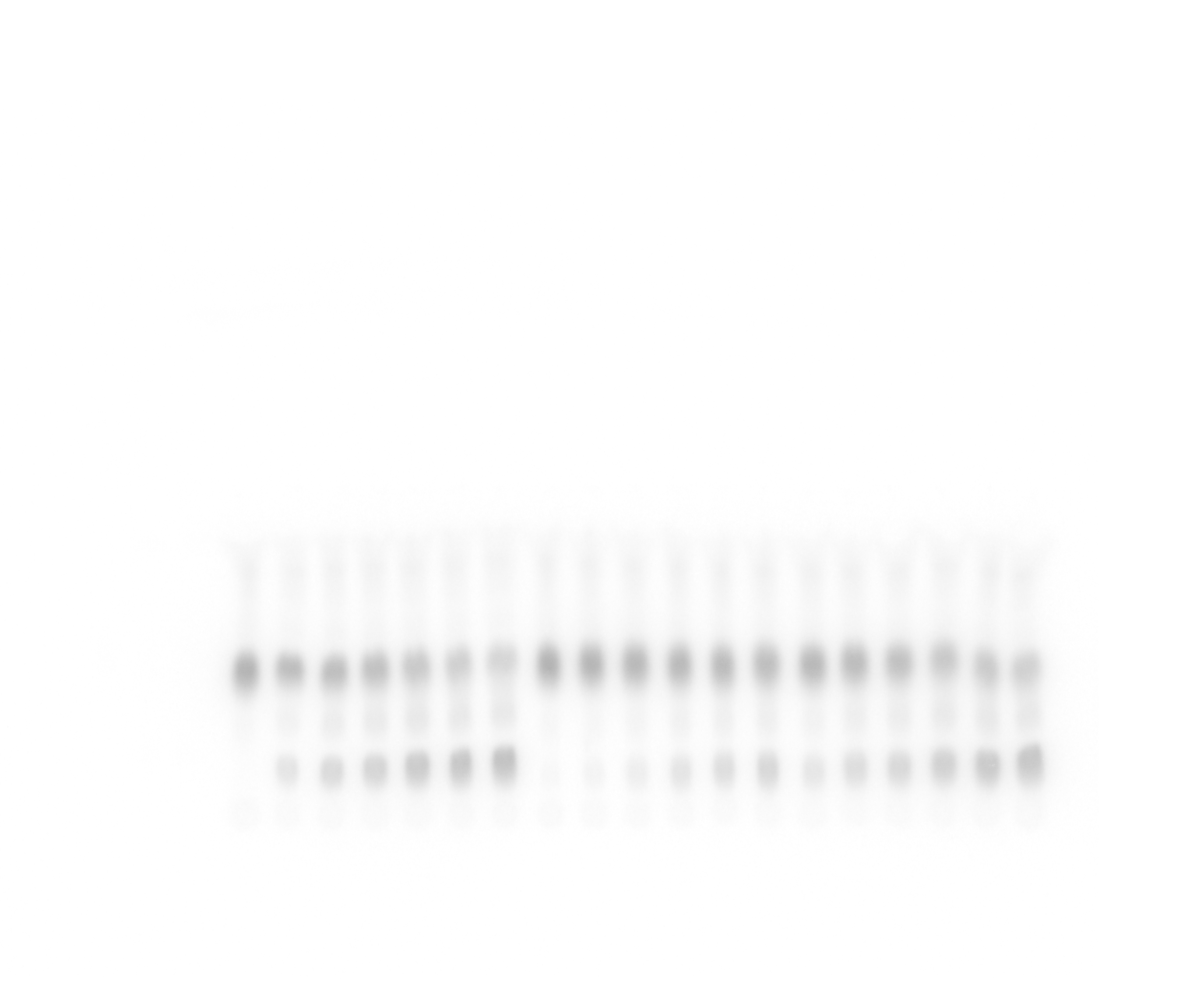

Supplement: Figure 6—source data 1. [file elife-64092-fig6-data1.zip › Figure 6-source data 1 (raw autoradiogram).gel]
