# Supplementary material for: NirD curtails the stringent response by inhibiting RelA activity in Escherichia coli
Source: eLife. 2021 Jul 29;10:e64092. doi: 10.7554/eLife.64092 (PMC8321558; doi:10.7554/eLife.64092)
Supplement: Supplementary file 1. [file elife-64092-supp1.docx]

**Supplementary File 1. Media used in this work**

| **Compounds** | **Quantities** |
| --- | --- |
| **Luria-Bertani (LB) broth** |  |
| Tryptone (Oxoid, LP0042B) | 10 g/L |
| Yeast extract (Oxoid LP0021B) | 5 g/L |
| NaCl | 10 g/L |
| **M9-glucose minimal medium** |  |
| Na_2_HPO_4_ | 60 mM |
| KH_2_PO_4_ | 22 mM |
| NaCl | 8 mM |
| NH_4_Cl | 20 mM |
| MgSO_4_ | 1 mM |
| CaCl_2_ | 100 µM |
| Thiamine | 1 µg/mL |
| Glucose | 0.2% |
| Agar bacteriological (Oxoid, LP0011) | 15 g/L |
| **Nutrient agar (NA)** |  |
| Nutrient agar (Oxoid, CM0003B) | 28 g/L |
| **SMG (M9-glucose minimal medium supplemented with** |  |
| L-Serine | 40 µg/mL |
| L-Methionine | 40 µg/mL |
| Glycine | 40 µg/mL |
| Agar bacteriological (Oxoid, LP0011) | 15 g/L |
| **Terrific broth (TB)** |  |
| Peptone | 1.2% |
| Yeast extract | 2.4% |
| K_2_HPO_4_ | 72 mM |
| KH_2_PO_4_ | 17 mM |
| Glycerol | 0.4% |
